# The *Aspergillus fumigatus maiA* gene contributes to cell wall homeostasis and fungal virulence

**DOI:** 10.3389/fcimb.2024.1327299

**Published:** 2024-01-26

**Authors:** Xabier Guruceaga, Uxue Perez-Cuesta, Adela Martin-Vicente, Eduardo Pelegri-Martinez, Harrison I. Thorn, Saioa Cendon-Sanchez, Jinhong Xie, Ashley V. Nywening, Andoni Ramirez-Garcia, Jarrod R. Fortwendel, Aitor Rementeria

**Affiliations:** ^1^ Department of Clinical Pharmacy and Translational Science, College of Pharmacy, University of Tennessee Health Science Center, Memphis, TN, United States; ^2^ Department of Immunology, Microbiology, and Parasitology, Faculty of Science and Technology, University of the Basque Country (UPV/EHU), Leioa, Spain; ^3^ Graduate Program in Pharmaceutical Science, College of Graduate Health Sciences, University of Tennessee Healths Science Center, Memphis, TN, United States; ^4^ Integrated Program in Biomedical Sciences, College of Graduate Health Sciences, University of Tennessee Health Science Center, Memphis, TN, United States; ^5^ Department of Microbiology, Immunology, and Biochemistry, College of Medicine, University of Tennessee Health Science Center, Memphis, TN, United States

**Keywords:** *Aspergillus fumigatus*, microarray, *maiA*, cell wall, virulence

## Abstract

In this study, two distinct *in vitro* infection models of *Aspergillus fumigatus*, using murine macrophages (RAW264.7) and human lung epithelial cells (A549), were employed to identify the genes important for fungal adaptation during infection. Transcriptomic analyses of co-incubated *A. fumigatus* uncovered 140 fungal genes up-regulated in common between both models that, when compared with a previously published *in vivo* transcriptomic study, allowed the identification of 13 genes consistently up-regulated in all three infection conditions. Among them, the *maiA* gene, responsible for a critical step in the L-phenylalanine degradation pathway, was identified. Disruption of *maiA* resulted in a mutant strain unable to complete the Phe degradation pathway, leading to an excessive production of pyomelanin when this amino acid served as the sole carbon source. Moreover, the disruption mutant exhibited noticeable cell wall abnormalities, with reduced levels of β-glucans within the cell wall but did not show lack of chitin or mannans. The *maiA-1* mutant strain induced reduced inflammation in primary macrophages and displayed significantly lower virulence in a neutropenic mouse model of infection. This is the first study linking the *A. fumigatus maiA* gene to fungal cell wall homeostasis and virulence.

## Introduction

Melanins are secondary metabolites composed of indolic monomers or phenolic polymers that act as pigments (Bayry et al., 2014). The saprophytic fungus *Aspergillus fumigatus* can produce two types of melanins. One of them is the dihydroxynaphthalene melanin, also known as DHN-melanin, which is one of the principal components of the conidial cell wall (Latgé and Chamilos, 2019) and together with the rodlet layer functions as the external hydrophobic layer of conidial cell wall. Underneath, forming the fibrillar layer are the β-(1,3)-glucans, chitin, α-(1,3)-glucans and galactomannan (Gastebois et al., 2009; Bayry et al., 2014; Beauvais and Latgé, 2015). When the conidia germinate, the external protective layer is shed, galactosaminogalactan is synthetized, and the fibrillar core is exposed to the environment. A second type of melanin is produced during mycelial growth, pyomelanin, which is a water-soluble pigment (Perez-Cuesta et al., 2020). This molecule is produced by spontaneous polymerization of homogentisate (HGA) during the L-phenylalanine/L-tyrosine degradation pathway (hereinafter named Phe degradation pathway). In this pathway, HGA is accumulated and converted to benzoquinone acetate by spontaneous oxidative transformation, then polymerized to pyomelanin. Finally, it is important to highlight that unlike other fungal species, such as *Cryptococcus neoformans* (Strycker et al., 2021) or even other *Aspergillus* species (Pal et al., 2013), *A. fumigatus* does not produce DOPA-melanin.

The principal function of DHN-melanin is protecting the fungus against environmental hazards such as desiccation (Gow et al., 2017), UVA, UVB and ionizing radiation (Eisenman and Casadevall, 2012; Braga et al., 2015). This pigment also has a protective role against amoeba predation (Hillmann et al., 2015), masks the *A. fumigatus* pathogen-associated molecular patterns (PAMPs) modulating monocytes and lymphocytes response (Luther et al., 2007; Chai et al., 2010), inhibits phagolysosome acidification (Jahn et al., 2002) and scavenges ROS production by host cells (Jahn et al., 1997). Furthermore, it has been demonstrated that the Δ*pksP/alb1 A. fumigatus* strain, white strain that no produces DHN-melanin, was less virulent than strains with normal production of this pigment (Jahn et al., 1997; Langfelder et al., 1998; Tsai et al., 1998). Among the biological functions that pyomelanin fulfills, this molecule seems to protect young hyphae against oxidative stress (Schmaler-Ripcke et al., 2009), and until now the importance of *A. fumigatus* pyomelanin for fungal virulence is still unclear (Perez-Cuesta et al., 2020).

Pyomelanin production is associated with conidial germination and directly depends on the cell wall integrity pathway (CWI) (Valiante et al., 2009). The CWI is a signaling cascade essential to maintain cell wall homeostasis (Valiante et al., 2015; Rocha et al., 2020; Ibe and Munro, 2021). This pathway is activated when cell surface sensors (Wsc1, Wsc3, and MidA) detect environmental stress that affect the fungal cell wall (Dichtl et al., 2012; Grice et al., 2013). This leads to activation of the Rom2 (Samantaray et al., 2013) and Rho1 complex. which, in turn, activates the apical kinase PkcA (Dichtl et al., 2012; Rocha et al., 2020). Activated PkcA subsequently phosphorylates the core CWI MAP kinase cascade, Bck1, Mkk2 and MpkA (Grice et al., 2013; Samantaray et al., 2013; Valiante et al., 2015; Rocha et al., 2020; Ibe and Munro, 2021). Once MpkA is phosphorylated it travels to the nucleus to phosphorylate RlmA that coordinates the response against stress (Rocha et al., 2020).

It has also been described that the *A. fumigatus* Phe degradation pathway is mediated by six genes located on chromosome 2 (*hppD*, *hmgX*, *hmgA*, *fahA*, *maiA* and *hmgR*). In addition, there are two other genes not included inside the abovementioned cluster, *phhA* and *tat*, which code for phenylalanine hydroxylase and the tyrosine aminotransferase, respectively (Heinekamp et al., 2013). They oversee the first degradation steps of Phe to Tyr, and Tyr to 4-hydroxyphenylpyruvate. There are several published studies uncovering the function of the genes of the Phe/Tyr degradation gene cluster and their importance for the fungus based on deletion/disruption mutants (Schmaler-Ripcke et al., 2009; Keller et al., 2011). However, there are still two genes of the cluster that are not well-characterized because they have not been silenced yet (*fahA* and *maiA*).

The *A. fumigatus maiA* is an exonic 696 bp gene (Afu2g04240) that does not contain introns in its sequence and codifies a maleylacetoacetate isomerase putatively involved in this Phe degradation pathway. Specifically, this enzyme orchestrates the isomerization of 4-maleylacetoacetate (4-MA) into 4-fumarylacetoacetate (4-FA) (Perez-Cuesta et al., 2020). In theory, the resulting phenotype of the defective *maiA* strain should phenocopy Δ*hmgA*, which is the gene that codifies the enzyme located just upstream of *maiA* in this degradation pathway.

In this study, the *A. fumigatus* genes most strongly over-expressed upon co-culture with two different cell lines (murine macrophages and human lung epithelial cells) were studied using the AWAFUGE microarray. Among them, *maiA* was selected because it was overexpressed in these two *in vitro* models, as well as in a previously published *in vivo* infection (Guruceaga et al., 2018). Therefore, to study its role in fungal biology and virulence, a disruption mutant strain (*maiA-1*) was generated and characterized. The data obtained strongly support the involvement of *maiA* in the maintenance of cell wall structure as well as in the virulence of the fungus using a neutropenic mouse model.

## Materials and methods

### *Aspergillus fumigatus* strains, media, and growth conditions

In this study, we used the *A. fumigatus* Af293 strain as the wild type genetic background to develop the disruption mutant strain *maiA-1* as well as its complement strain *maiA-1^comp.^* and the deletion mutant strain Δ*maiA*. All fungal strains were cultured in Glucose Minimal Media (GMM) (6 g NaNO_3_, 0.52 g KCl, 0.52 g MgSO_4_ x 7H_2_O, 1.52 g KH_2_PO_4_, 1 ml trace elements [2.2 g ZnSO_4_ x 7H_2_O, 1.1 g H_3_BO_3_, 0.5 g MnCl_2_ x 4H_2_O, 0.5 g FeSO_4_ x 7H_2_O, 0.16 g CoCl_2_ x 5H_2_O, 0.16 g CuSO_4_ x 5H_2_O, (NH_4_)_6_Mo_7_O_24_ x 4H_2_O, 5 g Na_4_EDTA in 100 ml distilled H_2_O], 10 g glucose, 15 g agar, pH 6.5, in 1 liter distilled H_2_O) at 37°C for five days, and their conidia were harvested directly from the agar plates using sterile water, followed by two washes with saline solution (0.9% NaCl). The concentration of conidia needed for each experiment was calculated using a Bürker counting chamber.

To study the ability of the fungal strains to grow with a sole carbon source, GMM was replaced by a GMM medium without glucose (named salt agar).

### Cell lines

The murine macrophage cell line RAW 264.7 and the human lung epithelium cell line A549, both obtained from the American Type Culture Collection (ATCC, Manassas, VA, USA), have been used in this study. The culture conditions as well as viability calculation and passage method were done following a previously described method (Guruceaga et al., 2021). Briefly, cultures were maintained in complete RPMI (RPMI 1640 supplemented with 10% heat-inactivated fetal bovine serum, 200 mM L-glutamine, 100 U/ml penicillin, and 0.1 mg/ml streptomycin) and incubated under standard conditions (37 °C, 5% CO_2_, and 95% humidity). Cell lines were used only when the viability of them after passage was higher than 90%. All supplies were purchased from Sigma-Aldrich (St. Louis, MO, USA).

### Phagocytosis assays and fungal behavior against RAW 264.7 cell line

To understand the behavior of the fungal strains in contact with the murine macrophages cell line RAW 264.7 we followed the method previously described (Guruceaga et al., 2021). Briefly, 2 x 10^5^ cells/ml in 500 µL of cell culture RPMI (RPMI 1640 medium supplemented with 10% heat-inactivated FBS, 200 mM L-glutamine, 100 U/ml penicillin and 0.1 mg/ml streptomycin) were seeded in 24-well plates which contained 12 mm-diameter coverslips. After overnight incubation, RAW264.7 cells were co-cultured with *A. fumigatus* conidia at a multiplicity of infection (MOI) of 10 (ten conidia per cell) using 500 µL of challenge RPMI (RPMI 1640 medium supplemented with 10% heat-inactivated FBS, and 200 mM L-glutamine). In parallel, we seeded the same amount of conidia in the absence of RAW 264.7 cells. After 2-, 4-, 6-, and 8-hours post-incubation we moved the coverslips to a new plate with cold PBS to calculate the percentage of phagocytosis, fungal germination, and hyphal branching using a Nikon Eclipse TE200-U inverted microscope.

### Endocytosis assays and fungal behavior against A549 cell line

To study fungal behavior in contact with the human alveolar epithelial cell line A549 we seeded 1 x 10^6^ cells/ml in 500 µL of cell culture RPMI using 24-well plates which contained 12 mm-diameter cover slips. After an overnight incubation, the cells were co-cultured with *A. fumigatus* conidia pre-stained with FITC (conidia were stained overnight at 4°C in an orbital shaker) at a MOI of 5 in 500 µL of challenge RPMI. At each incubation time (2, 4, 6, 8 and 10 hours), we moved the coverslip to a new plate to calculate the percentage of endocytosis. For that, a minimum of 500 cells/conidia/hyphae were counted in each replicate of the experiment using the inverted microscope Nikon Eclipse TE2000-U to calculate endocytosis (%), fungal germination (%) and hyphal branching (%) both in contact with epithelial cell lines and *A. fumigatus* growing alone as the control condition.

### RNA isolation and purification

For RNA isolation, we collected the cells and fungus using a cell scraper after the incubation time selected in each case (6.5 hours with RAW 264.7 and 8.5 hours with A549). Then, the samples were centrifuged for 1 minute at 14,000 rpm and the pellet was suspended in 1 ml of pre-chilled DEPC sterile deionized water to lyse the mouse/human cells. After that, the samples were centrifuged again in the same conditions abovementioned, the fungal pellet was suspended in 500 µL of pre-chilled DEPC-treated sterile deionized water and transferred to a 1.5 ml tube containing 200 µL of 0.5 mm glass beads (Sigma-Aldrich, St. Louis, MO). The samples were homogenized using the MillMix 20 beat-beater (Technica, Dulles, VA, USA) at 30 Hz for 2 minutes. Finally, we centrifuged the samples as mentioned above, and the supernatant was recovered and transferred to the extraction columns of the RNeasy Plant Mini Kit (Qiagen, Hilden, Germany). The RNA isolation procedure was finished following the manufacturer´s instructions and the RNA quantity and integrity was verified on a 2100 Bioanalyzer (Agilent Technologies, Santa Clara, CA, USA). For microarray analysis and RT-qPCR confirmation, three independent RNA samples for each time point, each of them obtained from an independent co-incubation with cells assays or controls, were studied.

### Microarray selection, hybridization, and expression data analysis

The fungal transcriptome was studied using the AWAFUGE microarray (Agilent Whole *A. fumigatus* Genome Expression 44K v.1). The microarray hybridization and the analysis of the raw data obtained was processed as previously described (Guruceaga et al., 2018). Raw intensity data was analyzed using the limma library implemented in the Bioconductor package (Smyth, 2004; López-Romero, 2011). Background subtraction and data normalizing was done using normexp and quantile routines respectively. We fixed a statistical significance level of 0.05 after analyzing the data using ANOVA test with Benjamini-Hochberg correction. Finally, we determined the genes down- or up-regulated relative to the fungal growth alone.

### Microarray data confirmation by reverse transcription quantitative PCR

We selected a subset of 22 genes to verify fungal expression profiles by RT-qPCR co-cultured with RAW 264.7 macrophages, and 22 genes with A549 pneumocytes. In both cases, we used the same 4 reference genes. Specific *A. fumigatus* primers were designed using Primers Quest Tool (available at “ eu.idtdna.com/site”) to avoid biased results due to mouse or human RNA remaining in the samples (**Table S1**). Selection of the most suitable housekeeping genes and analysis of the RT-qPCR results were completed following a previous publication (Guruceaga et al., 2018).

Finally, the expression levels of *uge3* and *pksP*, two essential genes in galactosaminogalactan and DHN-melanin biosynthetic processes, were studied by RT-qPCR assays following protocols previously published (Martin-Vicente et al., 2018).

### Gene ontology analysis

The GO enrichment of those DEGs (log FC > 1.5 or log FC < -1.5), was done using the Hans Knoell Institute FungiFun website (elbe.hki-jena.de/fungifun/) (Priebe et al., 2015).

### Gene target selection criteria

The selection of *maiA* as an important *A. fumigatus* gene involved in fungal virulence was determined after analyzing our transcriptomic results and previous transcriptomic studies (Guruceaga et al., 2018). We classified the genes following their fold change values (FC). We only focused our attention on those genes highly down- or up-regulated (FC < 1.5 or FC > 1.5 respectively). After that, we compared the common *A. fumigatus* up-regulated DEGs in three different assays: 1) *A. fumigatus* strain Af293 in co-incubation with lung epithelial cell line A549, 2) *A. fumigatus* strain Af293 in co-incubation with macrophages RAW 264.7 and 3) *A. fumigatus* strain Af293 infecting immunosuppressed mice (Guruceaga et al., 2018).

### Mutant strains generation

The *A. fumigatus* mutant strains used in this study were performed following the CRISPR/Cas9 strategy previously described (Al Abdallah et al., 2017). In this study, we used the Af293 genetic background to use the same strain used in the transcriptomic studies in which we selected *maiA* as interesting avoiding bias interpretations. Briefly, *A. fumigatus maiA* mutant strains were performed using Hygromycin B (Thermo Fisher, Waltham, MA, USA) as a selection marker. For the disruption mutant strain (*maiA-1*), Cas9 targets and the corresponding crRNA were designed to delete the initial methionine of the *maiA* gene. In contrast, for Δ*maiA* we designed two gRNAs located in the 5´UTR and 3´UTR respectively with the aim of replacing the whole target gene with the Hyg^R^ cassette. Transformation of *A. fumigatus* protoplasts was carried out following the classic protocol described in the literature (Yelton et al., 1984). Finally, the complement of the *maiA-1* strain (*maiA-1^comp.^*) was constructed by re-introducing the original *maiA* gene followed by the phleomycin resistance cassette in the native locus. All the primers and gRNAs used can be found in **Table S2**.

### Determination of the pyomelanin production

Pyomelanin production was measured following a previously published protocol (Schmaler-Ripcke et al., 2009). Briefly, GMM broth (150ml) was inoculated with 1 x 10^7^ conidia of each indicated *A. fumigatus* strain. After 20 h of pre-incubation, Phe or Tyr was added to a final concentration of 10 mM. Aliquots of 500 µL of GMM broth were taken at 24, 48 and 72 hours. Pigment formation was analyzed by direct absorbance measurements at 405 nm of the alkalized supernatants obtained by adding 20 µL of 5 M NaOH per ml of sample and centrifuging them at 16,000 x g for 2 min. Three independent replicates of the experiment were carried out on different days.

### *A. fumigatus* cell wall stress assay, spot dilution assay and radial growth

The cell wall stressor assays were done using 6-well plates (Invitrogen, Waltham, MA, USA). The wells were filled with GMM or SMM supplemented with increasing concentrations (20, 40, 60, 80, 120, and 160 µg/ml) of CR or CFW. In parallel, plates with GMM or SMM without stressors were used as controls. To study if chitin synthase activity was affected by the *maiA* gene silencing, GMM 6-well plates containing (0, 0.5, 1, 2, 4, and 8 µg/ml) of Nikkomycin Z were performed. To inoculate the plates, 5 µL of a 2 x 10^6^ conidia/ml stock of each strain was pipetted at the center of the plates. The pictures were taken after incubation of the plates at 37°C for 48 h.

The spot dilution assay of each strain was done following the method previously described (Martin-Vicente et al., 2018). Briefly, we inoculated 10^4^, 10^3^, and 10^2^ conidia (5 µL/drop) in GMM plates supplemented with 1.2M NaCl or 1.2M KCl as cell membrane stressor compounds or 5 mM Caffeine as a MAPK-inducing stress agent. To study the ability of the strains to use Phe, Tyr, or Phe/Tyr as the sole carbon source, we supplemented salt agar (GMM without glucose) plates with 50 mM Phe, 50 mM Tyr, or 50 mM Phe and 50 mM Tyr. The pictures were taken after incubation of the plates at 37°C for 48 h.

Furthermore, we characterized the radial growth ability of the *maiA-1* mutant strain compared to the Af293. For that, we seeded a 20 µL drop containing a suspension of 10^8^/ml fresh conidia in the middle of a GMM agar plate. The plates were incubated at 37°C for five days and the radial growth of the macroscopic colonies were measured daily.

Three independent replicates of the experiment were carried out on different days. Representative pictures of each condition are shown.

### Scanning electron microscopy

For the cell wall surface study, 5 x 10^5^ conidia/ml from the Af293 and *maiA-1* strains were seeded in 1 ml of GMM in 24-well plates containing glass coverslips. The plates were incubated at 37°C, 5% CO_2_ and 95% of humidity for 12 hours. The fungal surface of each strain was studied after 2, 4, 8 and 12 hours of incubation by SEM. At each timepoint indicated, the GMM was removed, and fixing solution (2% glutaraldehyde in PBS buffer) was added to each well for 1h at 4°C. Then the samples were dehydrated through increasing ethanol concentrations and hexamethyldisilane. Finally, they were covered with gold under an argon atmosphere, and visualized under the scanning electron microscope (Hitachi S-4800).

### Mouse bone marrow macrophages isolation and cytokine measurements

BMMs were isolated following a previous publication (Pellon et al., 2018). The *in vitro* challenge of the BMMs was done following the same conditions previously described for RAW 264.7 cells. TNF cytokine determination was doing using the mouse TNF-αuncoated ELISA kit (Invitrogen, Waltham, MA, USA) following manufacturer’s instructions after 16 hours of co-incubation between BMMs and *A. fumigatus*.

### Fungal susceptibility to H_2_O_2_ oxidative agent

The susceptibility of the fungal strains to H_2_O_2_ was measured using GMM agar plates following a diffusion assay. Briefly, conidia of both strains (1 x 10^7^) were seeded and spread evenly over the surface of a GMM agar plate (90 mm petri dishes). Once the fungus was seeded, a central well was generated in the middle of the agar plate using a sterile pipet tip. Finally, 50 µL of a 200 mM solution of H_2_O_2_ was added to the central well. After drying for 5 minutes, the plates were incubated at 37°C for 48 hours. Three independent replicates of the experiment were carried out on different days.

### Study of cell wall components

The β-glucan assay was performed following methods previously described (Fortwendel et al., 2009; Koenig et al., 2017; Souza et al., 2019). Briefly, 1 x 10^7^ conidia of each strain were grown overnight into 25 ml of GMM broth at 37°C. After 16 hours of growing, hyphae were collected by filtration through Miracloth (Sigma-Aldrich, St. Louis, MO, USA) and washed using 0.1 M NaOH solution. Washed fungal hyphae were lyophilized for 24 hours. Five milligrams of dry hyphae were disrupted in a bead-beater three times (1 minute each) with 1 minute of ice incubation between each. Hyphal powder was resuspended to a final concentration of 20 mg/ml in 1 M NaOH and the solution was incubated at 52°C for 30 min. Fifty microliters of each sample were mixed with 185 µL of aniline blue staining solution (183 mM glycine, 229 mM NaOH, 130 mM HCl, and 618 mg/l aniline blue, pH 9.9) into a 96-well masked fluorescence plate (Invitrogen, Waltham, MA, USA). The sample-containing plates were incubated at 52°C for 30 min, followed by a cool down period of 30 min at room temperature. Fluorescence readings were performed using an excitation/emission wavelength of 405/460 nm respectively. All the experiments were performed in triplicate using three independent *A. fumigatus* cultures, and the results were represented as relative quantification versus the Af293 strain.

Chitin content analysis was performed following protocols previously published (Cánovas et al., 2017; Rocha et al., 2020). Briefly, 96 well plates containing 100 µl of GMM per well were inoculated with 10^3^ conidia/well of each strain and incubated at 37 °C for 16, 18, 20, 22, and 24 hours. After each incubation time, 150 µl of CFW stock solution (10 µg/ml in cell culture PBS) was added to each well. Fluorescence (excitation, 360 nm; emission, 460nm) and absorbance (600 nm) were measured in parallel in a BioTek multiplate reader. The slope, calculated fluorescence against absorbance, of each strain was plotted. The experiment was repeated eight times with technical replicates.

Fluorescence microscopy study of cell wall chitin, mannans and exposed chitin of the fungal strains was completed using CFW (1 µg/ml in cell culture PBS), concanavalin Alexa Fluor 647 conjugate (Invitrogen, Waltham, MA, USA), and Wheat Germ agglutinin Alexa Fluor 633 conjugate (Invitrogen, Waltham, MA, USA), respectively. To do this, 24-well glass bottom plates were inoculated with 1 x 10^3^ conidia and incubated for 16 hours at 37 °C. After the incubation time, samples were observed under Nikon Eclipse T*i*2.

### Murine model of invasive pulmonary aspergillosis

Groups of 20 female CD-1 mice (Charles River, Wilmington, MA, USA), weighing approximately 25 g, were immunosuppressed by intraperitoneal injections of 150 mg/kg cyclophosphamide (Sigma-Aldrich, St. Louis, MO) starting 4 days before the inoculation and every 3 days following, using 75 mg/kg of cyclophosphamide, and a single subcutaneous injection of 40 mg/kg triamcinolone acetonide (Kenalog, Bristol-Myers Squibb) 24 hours before the infection. On day 0, mice were transiently anesthetized with isoflurane and challenged via nasal inoculation with 10^6^ conidia in sterile saline solution. Survival was monitored at least twice a day and those animals showing severe signs of distress were humanely euthanized by anoxia with CO_2_. Survival curves were compared using the log-rank test in GraphPad Prism v. 8.2.1 for Windows. In parallel, histological studies were carried out to understand if loss of *maiA* impacts over the early infection establishment. To do this, 4 mice were infected with Af293, and 4 mice were infected with the *maiA-1* strain, following the same protocol and conditions previously explained. At 3 days post-infection, mice were euthanized, and their lungs were harvested and fixed in 10% buffered formalin for 72 hours before being paraffin embedded. Subsequently, 4 µm slices were sectioned, mounted, and stained with hematoxylin-eosin (H&E) and Grocott’s Methenamine Silver (GMS). The studies were performed in accordance to approved ethical protocols by the LACU committee of the University of Tennessee Health Science Center (Protocol number 22-0373).

### Statistics

All the assays were done in triplicate on three independent days. All the statistical analysis were carried out using GraphPad Prism v. 8.2.1 (GraphPad Software Inc., San Diego, CA, USA) for Windows. In each assay, at least three biological replicates were done to measure each parameter in each condition, avoiding biased results. Error bars included in all the graphs represent standard deviation. ANOVA or t-test was used depending on if we did multiple comparisons or compared punctual data, respectively, after ensuring the data sets followed a normal distribution.

### Availability of data

The ArrayExpress database (www.ebi.ac.uk/biostudies/arrayexpress) contains the AWAFUGE microarray (v.1) design under accession number A-MEXP-2352. The same database also contains each raw microarray dataset obtained under accession number E-MTAB-13476. Moreover, the *in vivo* transcriptomic analysis previously published can be found in the same database under accession number E-MTAB-5314.

## Results

### Study of co-incubation between *A. fumigatus* and macrophages

**Figure 1:**
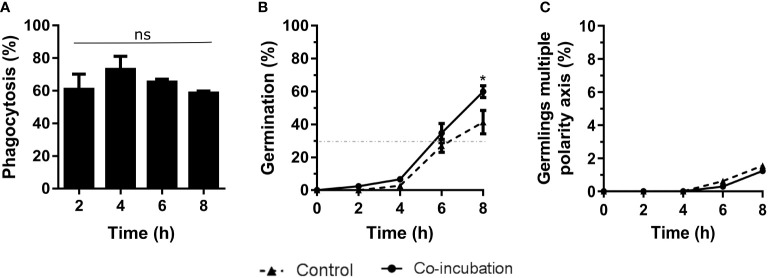
Characterization of the co-incubation between the fungal strain Af293 and the macrophage cell line RAW 264.7. **(A)** Phagocytosis assay during 8 hours of co-incubation with Af293. To do that, 500 µL of challenge RPMI containing 2 x 10^5^ macrophages and *A*. *fumigatus* conidia in a MOI of 10 were seeded in 24-well plates which contained 12 mm-diameter coverslips. **(B)** Percentage of germination of the Af293 strain alone and in with co-incubation with RAW 264.7 macrophages. The dashed line indicates 30% germination. **(C)** Percentage of Af293 germlings with multiple polarity axes alone and in co-incubation with RAW 264.7 macrophages. Statistical analysis in panel **(A)** was performed by One-Way ANOVA followed by multiple comparisons. ns p > 0.05. Statistical analysis in panel **(B, C)** were performed by t-test. *p < 0.05.

The results obtained from co-incubation with the murine macrophage RAW 264.7 cell line revealed 80% phagocytosis after 4 hours of co-incubation (**Figure 1A**), but no statistical differences were observed. Although the germination of conidia reached 30% at 6 hours in all cases (infection and control cultures), at the end of the experiment significantly higher germination was detected in the conidia incubated with macrophages, compared to the controls (**Figure 1B**). In contrast, we did not find any differences in the amount of hyphal growth or their ability to stablish a second axis of polarity during germination (**Figure 1C**).

### Study of co-incubation between *A. fumigatus* and epithelial cells

**Figure 2:**
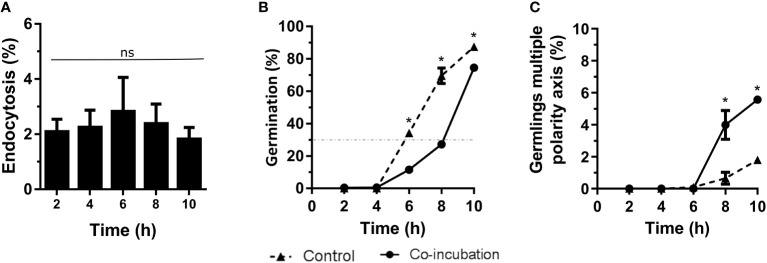
Characterization of the co-incubation between the fungal strain Af293 and the human epithelial cell line A549. **(A)** Endocytosis assay during 10 hours of co-incubation with Af293. To do that, 500 µL of challenge RPMI containing 1 x 10^6^ macrophages and *A. fumigatus* conidia in a MOI of 5 were seeded in 24-well plates which contained 12 mm-diameter coverslips. **(B)** Percentage of germination of Af293 in co-incubation with the lung epithelial cell line A549 in the same conditions explain in panel **(A)**. The dashed line indicates 30% germination. **(C)** Percentage of Af293 germlings with multiple polarity axes alone and in co-incubation with the lung epithelial cell line A549 in the same conditions explain in panel **(A)**. Statistical analysis in panel **(A)** was performed by One-Way ANOVA followed by multiple comparisons. ns p > 0.05. Statistical analysis in panel **(B, C)** were performed by t-test. *p < 0.05.

Co-incubation of *A. fumigatus* with the lung epithelial cell line, A549, revealed a maximum endocytosis of approximately 5% after 6 hours of co-incubation (**Figure 2A**). In addition, the contact with these epithelial cells generated a slight inhibition of the germination rate. Indeed, 30% of germination was reached after 8 hours of co-incubation with the epithelial cells while this germination rate was reached at 6 hours when the fungus grew alone (**Figure 2B**). However, an increase in germlings with multiple polarity axis was observed after 8 hours of co-incubation of *A. fumigatus* with epithelial cells (**Figure 2C**).

### *A. fumigatus* gene expression in response to the co-incubation with RAW 264.7 macrophage or A549 epithelial cell lines

To understand the molecular changes that *A. fumigatus* suffers in the onset of the infection process, we consider 30% of germination a suitable endpoint for this purpose because in this time point both the germlings and the remaining swollen conidia are starting the activation and adaptation of their metabolism for infection. This happened after 6 and 8 hours of co-incubation with macrophages and epithelial cells, respectively, moment in which three samples of mRNA from each condition obtained in independent experiments, as well as their respective controls, were hybridized with the AWAFUGE microarray. Once the data were analyzed and normalized, 2,137 and 5,325 *A. fumigatus* genes were found differentially expressed with respect to their controls (*A. fumigatus* without cells) when the fungus was incubated with macrophages and with human lung epithelial cells, respectively.

**Figure 3:**
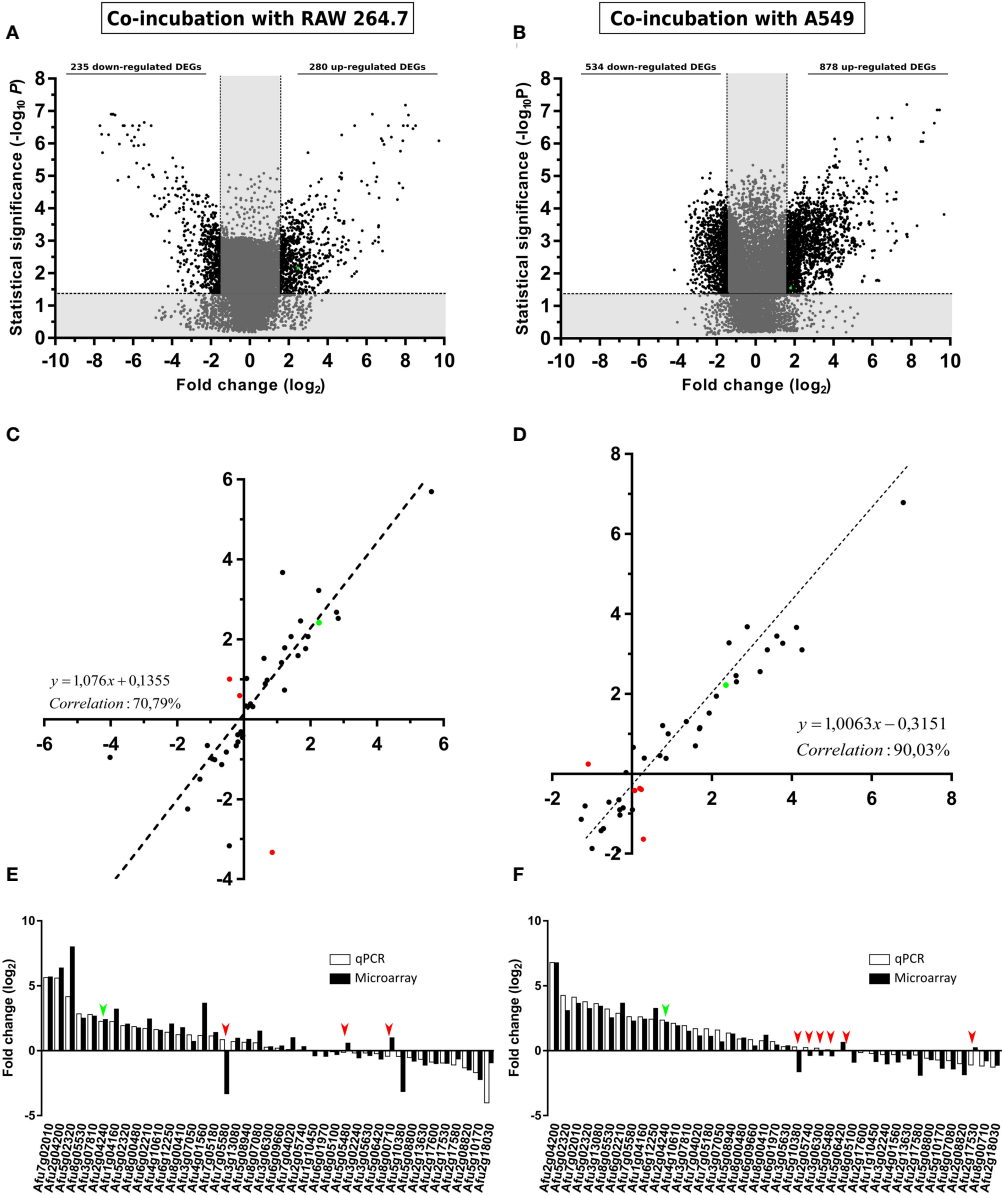
Gene expression analysis. Volcano plot showing *A. fumigatus* differentially (black) (FC > 1.5 or FC < -1.5) and non-differentially (grey) expressed genes in co-incubation with RAW 264.7 macrophages **(A)**, A549 lung epithelial cells. **(B)**. The x-axis values represent the fold change (log_2_) of microarray data and the y-axis values represent the statistical significance (–log_10_P). Spots with positive values indicate upregulation of the gene during co-incubation with the indicated cell line. **(C, D)** Correlation analysis between microarray and RT-qPCR data. The x-axis values represent the fold change (log_2_) of microarray data and the y-axis values represent the fold change (log_2_) of RT-qPCR results of the selected fungal genes in co-incubation with RAW 264.7 macrophages **(C)**, and A549 lung epithelial cells **(D)**. Each point corresponds to the mean value from three independent samples. **(E)** Comparative levels of fungal gene expression between the microarray and the RT-qPCR (Af293 in co-incubation RAW 264.7 macrophages). **(F)** Comparative levels of fungal genes expression between the microarray and the RT-qPCR (Af293 in co-incubation with A549 lung epithelial cells). Green points in **(A–D)** and green arrows in **(E, F)** show the corresponding results obtained for *maiA*. Red spots in **(C, D)** and red arrows in **(E, F)** show contradictory results in gene expression detected between microarrays and RT-qPCR results.

To limit and prioritize the genes to be studied, only those displaying > 1.5 or < -1.5-fold change (log_2_) (FC) in their conditions were considered. By this criteria, 235 A*. fumigatus* genes were down-regulated, and 280 genes were up-regulated during the co-incubation with the macrophages (**Figure 3A**), whereas 534 were down-regulated and 878 genes were up-regulated during the contact with the epithelial cells (**Figure 3B**).

The transcriptomic data obtained from the AWAFUGE microarray was confirmed by RT-qPCR, showing a good correlation result between both techniques. Specifically, a correlation of 70.79% and 90.03% was obtained between microarray and RT-qPCR verification in the co-incubation of *A. fumigatus* with macrophages, and with epithelial cells, respectively (**Figures 3C, D**). In **Figures 3E, F**, FC values obtained for each gene and technique used in the validation process are shown. All of them, except those marked in red (dots in panels C and D and arrows in panels E and F), displayed a similar expression in both techniques. In addition, *maiA* gene expression values were plotted in green.

### GO enrichment analysis of the most up/down-regulated DEGs and comparison between different infection models

To identify those *A. fumigatus* processes, components, and functions of greatest importance that were impacted during the experimental infection procedure, we performed a Gene Ontology (GO) enrichment analysis (the complete analysis of DEGs can be found in **Table S3**).

Among the DEGs, 227 (RAW 264.7 vs Control) and 592 (A549 vs Control) were associated with a known biological process. The five most relevant processes in this category are those related with transport, regulation, response to stress, secondary metabolic process, and lipid metabolic process.

In addition, 252 A*. fumigatus* genes (RAW 264.7 vs Control) and 605 genes (A549 vs Control) were associated with a known molecular function. The categories corresponding to the most genes are oxidoreductase activity, hydrolase activity, and transporter and transferase activities.

Moreover, it is remarkable that 321 (RAW 264.7 vs Control) and 795 (A549 vs Control) genes have been previously associated with a known cellular location, including (in order of gene numbers associated) membrane, mitochondrion, nucleus, cytosol, plasma membrane and the extracellular region.

To select the most important genes related to infection, among the large amount of overexpressed *A. fumigatus* genes found, those up-regulated with a FC > 1.5 in co-incubation in common between macrophages and epithelial cells were selected. Using this approach, a total of 140 A*. fumigatus* genes were obtained (**Table S4**). These genes were then cross-referenced with those detected as overexpressed during a previous mouse infection model (Guruceaga et al., 2018). This analysis decreased the list to 13 significantly up-regulated summarized in **Table 1**. Among those, Afu2g04240 (*maiA*) gene was selected as a good candidate to be characterized in this study for its role in *A. fumigatus* for the next reasons: i) It is one of the genes related with the Phe degradation pathway still unstudied, ii) Its potential role in the infection process detected in our transcriptomic analysis.

**Table 1:** Common overexpressed DEGs in the three experimental infection models.

			Fold Change (log_2_)		
ID	Product		RAW 264.7 vs Control	A549 vs Control	Intranasal infection (Day 4 vs Day 1) ^a^
Afu1g04160	Aspartate aminotransferase		3.22	2.45	3.22
Afu2g04200	4-hydroxyphenylpyruvate dioxygenase		6.38	6.78	4.21
Afu2g04240	Maleylacetoacetate isomerase	*maiA*	2.42	2.22	3.72
Afu3g07810	Succinate dehydrogenase subunit	*sdh1*	2.72	1.52	2.90
Afu4g10610	Stress responsive A/B barrel domain protein		1.59	1.94	4.39
Afu5g02320	Conserved hypothetical protein		2.07	3.27	3.33
Afu5g02330	Major allergen and cytotoxin Asp f 1	*aspf1*	3.00	3.10	4.64
Afu6g00430	IgE-binding protein		2.84	2.24	4.02
Afu6g02210	Cytochrome P450 monooxygenase		2.46	3.68	4.28
Afu6g03590	Citrate synthase	*cit1*	2.61	3.54	4.05
Afu6g12250	Succinyl-CoA:3-ketoacid-coenzyme A transferase putative		2.07	3.28	3.61
Afu7g02010	Indoleamine 2,3-dioxygenase family protein		5.70	3.66	5.72
Afu8g05530	Fumarate reductase	*osm1*	2.52	2.56	3.97

^a^Data obtained from a previous publication (Guruceaga et al., 2018 **).**

### The *maiA* gene is essential for Phe degradation pathway

**Figure 4:**
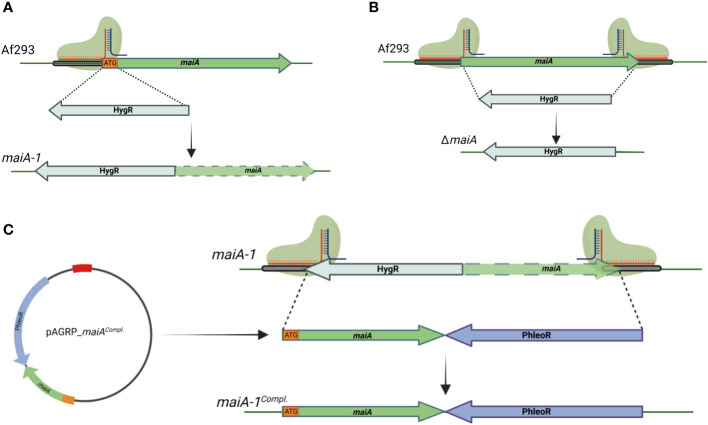
Schematic of gene manipulations by CRISPR/Cas9 editing. **(A)** Silencing strategy of the *maiA* gene. One protospacer adjacent motif was designed to disrupt the iMet (ATG orange square) of the *maiA* gene. The disruption was repaired using the hygromycin resistance cassette with 40 base pair microhomology regions upstream and downstream the *maiA* gene in the Af293 genetic background to generate the *maiA-1* mutant strain (*maiA* green dashed arrow represents non-functional *maiA* gene). **(B)** The complete deletion of the *maiA* gene was carried using two protospacers adjacent motif designed to the flanking regions of the *maiA* gene. The repair template process was developed following the same strategy described in panel **(A)** to generate the Δ*maiA* mutant strain. **(C)** To complement the *maiA-1* mutant strain, the native *maiA* gene was cloned into pAGRP plasmid (iMet included) upstream the phleomycin resistant cassette and generating the pAGRP_*maiA^comp.^* plasmid. Two protospacers adjacent motif designed to the flanking regions of the *maiA* gene were used. The repair template cassette was amplified from the pAGRP_*maiA^comp.^* plasmid using primers containing 40 base pair microhomology regions upstream and downstream the HygR-*maiA* disrupted regions, generating the *maiA-1^comp.^* mutant strain. All the colonies were confirmed by PCR and sequencing. The genetic maps were created with BioRender.com.

The genetic manipulation strategy followed to generate the mutant strains is described in Materials and Methods section and summarized in **Figure 4**. Briefly, a disruption mutant was generated by replacing the initial methionine (iMet) with the hygromycin resistance gene. The resulting mutant strain (*maiA-1*) was resistant to hygromycin and has the target gene silenced due to the lack of the iMet (**Figure 4A**). The complete deletion strain (Δ*maiA*) was also performed, through complete substitution of the *maiA* locus by the hygromycin resistance gene (**Figure 4B**). Finally, the complement of the *maiA-1* strain (*maiA-1^comp.^*) was constructed by replacing the disrupted allele with the native *maiA* followed by the phleomycin resistant gene in the native locus (**Figure 4C**). All the strains were generated using CRISPR-Cas9 highly efficient genetic technology in the Af293 genetic background. The Δ*maiA* strain was constructed to validate the results obtained in the disruption strain. As is possible to see in (**Figures S1** and **2**) the deletion strain phenocopied the *maiA-1* strain results, confirming the usefulness of the disruption strategy.

**Figure 5:**
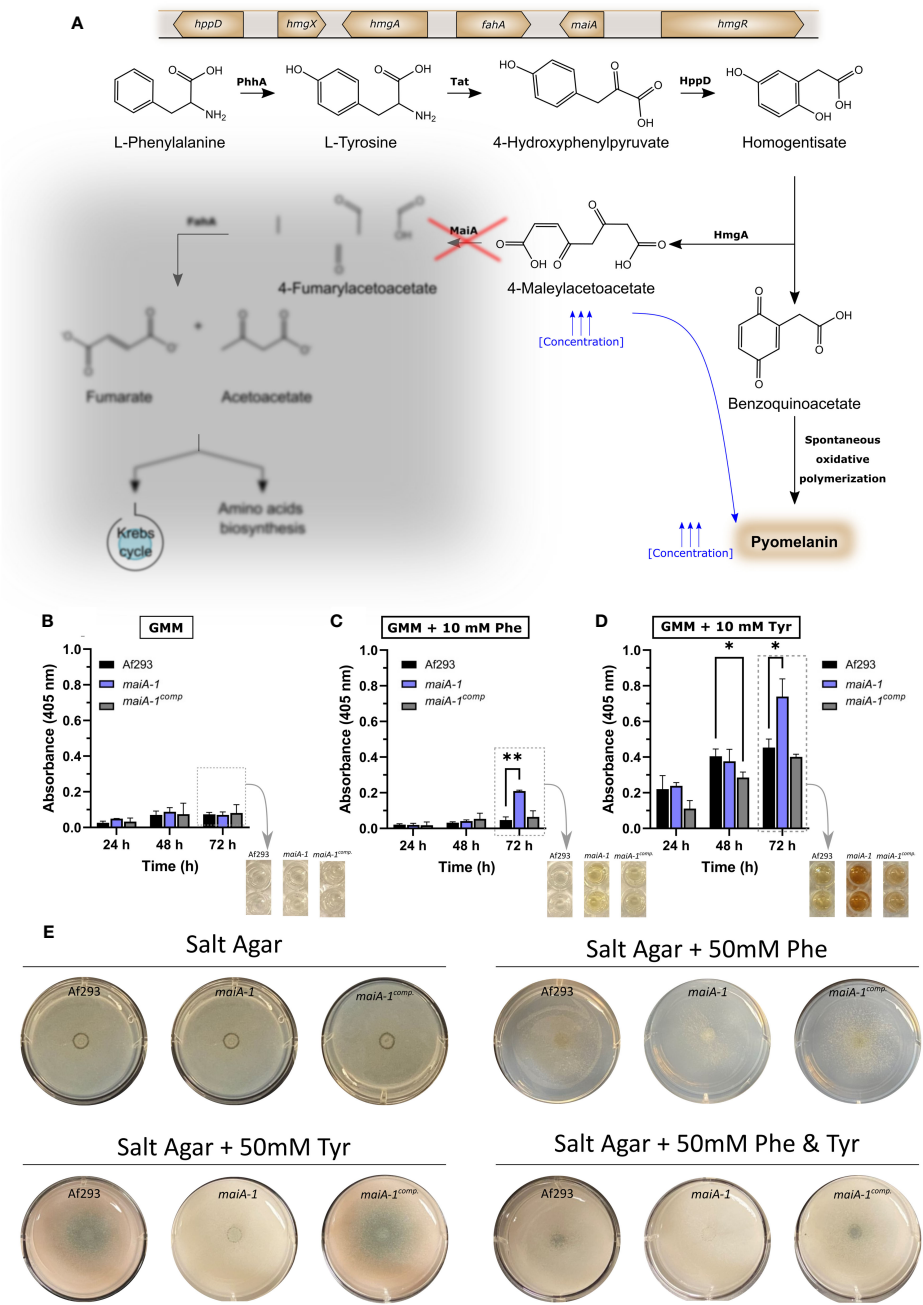
Fungal strain characterization results in relation to pyomelanin production and growth ability in presence of Phe and/or Tyr. **(A)** Phe degradation pathway including the genetic cluster and the proteins involved in each step. The red cross indicates the point in which the pathway is disrupted due to the disruption produced in *maiA-1* strain. The diffused grey area indicates the part of the pathway that the *maiA-1* strain cannot perform. Pyomelanin secretion time course of Af293, *maiA-1*, and *maiA-1^comp.^* strains growing for 72 hours in **(B)** GMM broth, **(C)** GMM broth supplemented with 10 mM Phe and **(D)** GMM broth supplemented with 10 mM Tyr. Dashed boxes in **(B–D)** indicate quantitative information corresponding to the photographs indicated by arrows. Statistical analyses were performed by Two-way ANOVA with Tukey’s test for multiple comparisons. *p < 0.05 or **p < 0.001. **(E)** Ten thousand fresh conidia of the Af293, *maiA-1*, and *maiA-1^comp.^* strains were inoculated onto salt agar plates or salt agar plates in which the only carbon source was Phe (50 mM), Tyr (50 mM), or Phe and Tyr (50 mM each). Plates were incubated at 37°C for 72h. Note that *maiA-1* mutant showed a non-significant basal growth probably produced by residual sugars present in the agar. No growth was detected when the assay was done in liquid media following the same conditions (Data not shown).

As mentioned above, we hypothesized that loss-of-function mutations at the *maiA* locus would produce high concentrations of 4-MA that could polymerize spontaneously to pyomelanin (**Figure 5A**). To study the pyomelanin production ability, Af293 as well as the *maiA-1* mutant and the *maiA-1^comp.^* strains were grown for 72 hours in GMM broth (**Figure 5B**), GMM broth supplemented with 10 mM of Phe (**Figure 5C**) or GMM broth supplemented with 10 mM of Tyr (**Figure 5D**). Indeed, when Phe or Tyr were added to the media, the *maiA-1* mutant strain accumulated pyomelanin indicated by an increase of the 405 nm absorbance signal (**Figure 5C**) that can be also visually observed (**Figure 5D**). A varying ability to degrade both amino acids was shown since the production of pyomelanin was different when Phe or Tyr were used, likely because Phe may be utilized by other metabolic pathways, compared to Tyr (**Figure S3**).

It was also striking that when salt agar plates were supplemented with 50 mM of Phe, 50 mM of Tyr or 50 mM of both amino acids as the sole carbon source, the *maiA-1* strain exhibited impaired growth (**Figure 5E**). Attempts to measure biomass accumulation in submerged culture to further quantify the reduced ability to utilize these amino acids as carbon sources revealed a complete lack of growth for the *maiA-1* mutant in both Phe and Tyr (data not shown).

### Role of *maiA* in growth and development of *A. fumigatus*

**Figure 6:**
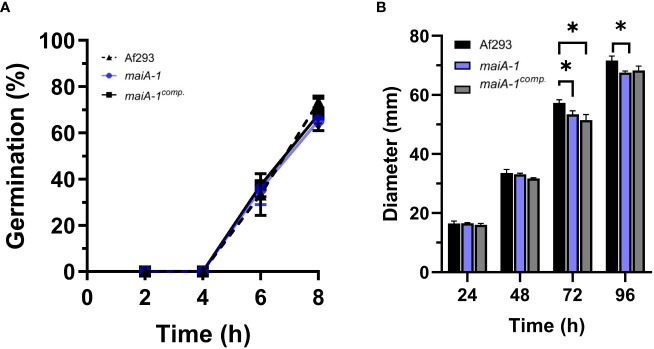
Cellular characterization results of the *maiA-1 mutant* strain. **(A)** Percentage of germination during 8 hours of incubation in GMM of the Af293, *maiA-1*, and *maiA-1^comp.^* strains. **(B)** Colony diameters of 2 x 10^6^ fresh conidia of each strain (Af293, *maiA-1*, and *maiA-1^comp.^*) seeded onto GMM plates. The diameter of each strain was measured daily for a maximum of 5 days. Statistical analyses were performed by two-way ANOVA with Tukey’s test for multiple comparisons. *p< 0.05.

To understand the role and importance of the *maiA* gene in the basal growth of *A. fumigatus*, the *maiA-1* disrupted strain was analyzed using germination and radial growth assays in GMM). Although the germination rate of all the strains was the same over the time studied (**Figure 6A**), slight differences in the radial growth ability were found between the Af293, the *maiA-1* and *maiA-1^comp.^* after 72 hours of growth. After 96 hours, no difference in the radial growth between Af293 and the complemented strain was found. In contrast the *maiA-1* mutant strain colonies were slightly smaller compared to those from the Af293 background (**Figure 6B**). Although statistical differences exist at the various timepoints, no overt defects in colony formation were seen.

**Figure 7:**
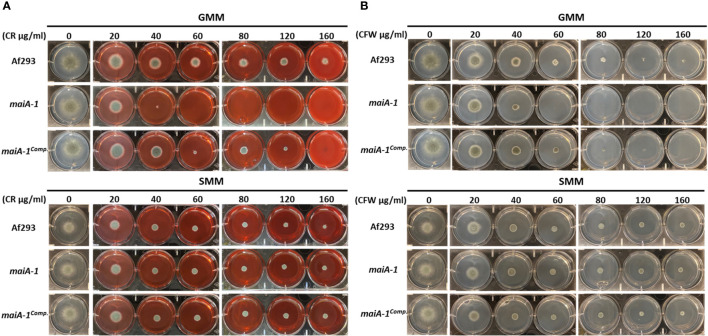
Phenotypic characterization of Af293, *maiA-1*, and *maiA-1^comp.^* strains using GMM agar 6-well plates. GMM or SMM 6 well plates supplemented with different concentrations (0, 20, 40, 60, 80, 120, 160 µg/ml) of congo red (CR) **(A)** or calcofluor white (CFW) **(B)** were inoculated using 1 x 10^3^ fresh conidia of each strain (Af293, *maiA-1*, and *maiA-1^comp.^*). The plates were incubated at 37°C for 48 h, time in which pictures were taken. Identical control wells are demonstrated in the two upper panels for GMM alone without the addition of either CR or CFW, and identical control wells are demonstrated in the two lower panels for SMM alone. Representative images of 3 replicates per condition are shown.

To better understand the role of *maiA* in maintaining fungal cell wall homeostasis, a stress response assay using two cell wall stressor compounds was performed (**Figure 7**). The *maiA-1* mutant strain was hyper-susceptible to the cell wall stressors congo red (CR) and calcofluor white (CFW) as observed by the inability to grow at concentrations higher than 40 µg/ml of both compounds in GMM (**Figure 7** top panels). The ability of this mutant strain to grow in the presence of both compounds was recovered by the addition of sorbitol to the media (SMM) (**Figure 7** bottom panels), further supporting that growth inhibition in the mutant was due to cell wall instability.

The CFW results suggest that *maiA-1* mutant could present some lack of chitin in its cell wall. However, as can be seen in **Figure S4**, the ability of the strains to grow in presence of Nikkomycin Z indicates that chitin synthase activity is not affected in the disruption mutant (**Figure S4A**). In addition, the abundance of chitin between fungal strains is the same (**Figure S4B**), a finding that was corroborated by fluorescent microscopy using CFW as a chitin indicator dye (**Figure S4C**). All these results reject the hypothesis of reduced chitin in the mutant cell wall.

### The *maiA-1* hyphae display an unstructured morphology

**Figure 8:**
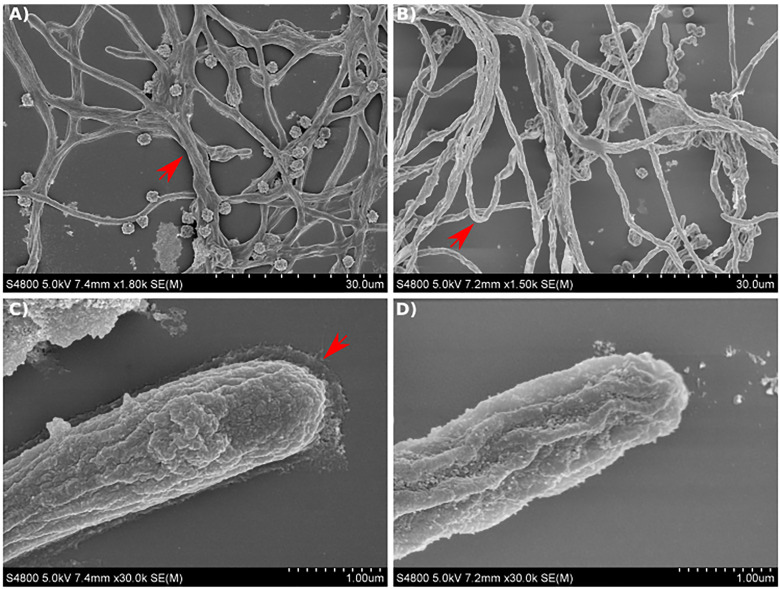
Scanning electron micrographs of *A. fumigatus* strains growing in GMM broth for 12 hours. **(A, C)** SEM images of Af293 hyphae. **(B, D)** SEM images of *maiA-1* hyphae. Red arrows denote specific details described in text. Representative images of 3 replicates per condition are shown.

Scanning electron microscopy (SEM) analysis of the mycelial appearance after culturing for 12 hours in GMM broth showed that the Af293 strain maintained a normal hyphal morphology with typical 45° branch angles and mycelia that were strongly attached to the surface (**Figure 8A**). In addition, analyzing the images at higher magnification, the Af293 hyphae were surrounded by an extracellular matrix, which was more prevalent at the hyphal apex (**Figure 8C**).

On the contrary, the *maiA-1* strain displayed a disorganized mycelium, which seemed to be more aerial, less attached to the surface and in consequence more rounded (**Figure 8B**) without the surrounding matrix observed in Af293 (**Figure 8D**).

Furthermore, other cell wall components such as N-acetylglucosamine exposed residues (exposed chitin) and mannans were studied through fluorescence microscopy, and no differences either in amount (changes in fluorescence intensity) or in cell distribution along the hyphae were detected (**Figures S5A, B**). While chitin is more exposed in the hyphal tips and around the conidia where the first germ tube was formed (**Figure S5A**), mannan is homogenously distributed along the hyphae (**Figure S5B**) of all the fungal strains studied. Although the SEM findings could imply a lack of galactosaminogalactan due to the loss of *maiA* function, no differences in expression levels of *uge3* between fungal strains were detected (**Figure S6A**). Furthermore, with the aim to exclude over production of DHN-melanin as a compensatory mechanism due to lack of *maiA*, expression levels of the *pksP* gene were measured in the fungal strains. As is possible to observe (**Figure S6B**), no expression differences of the *pksP* gene were detected between our fungal strains.

### *maiA* is required for normal *A. fumigatus* virulence

**Figure 9:**
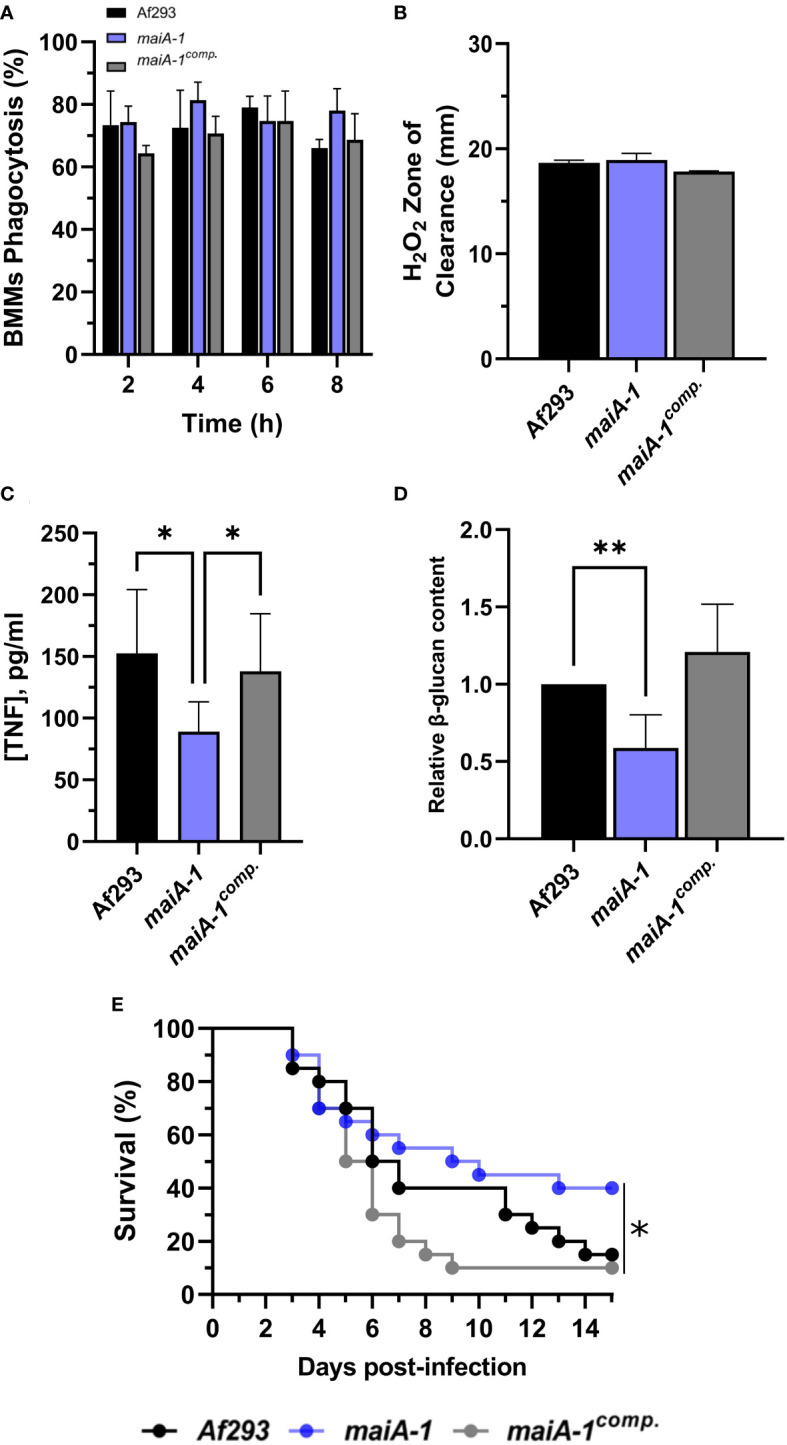
Characterization of the Af293, *maiA-1*, and *maiA-1^comp.^* strains during the infection process. **(A)** Phagocytosis measured after 8 hours of co-incubation between the three fungal strains and primary BMMs. To do that, 500 µL of challenge RPMI containing 2 x 10^5^ BMMs and *A. fumigatus* conidia in a MOI of 10 were seeded in 24-well plates which contained 12 mm-diameter coverslips. **(B)** Inhibition halus diameter of a culture of the three fungal strains on GMM agar plates after 48 hours of incubation in presence of 200 mM H_2_O_2_ in a central well of the plate. To do that, 1 x 10^7^ fresh conidia of each strain were seeded and spread over a GMM aga plate. After that, 50 µL of a 200 mM solution of H_2_O_2_ was added into a central well previously generated. The plates were incubated at 37°C for 48h. **(C)** BMMs TNF release after 16 hours challenge with the three fungal strains following the conditions described in panel **(A)**. **(D)** Relative content of β-glucans quantified in the three fungal strains. **(E)** Kaplan-Meier analysis of chemotherapeutically immune suppressed infected mice for 15 days post intranasal exposition to Af293, *maiA-1*, and *maiA-1^comp.^* fungal strains. Statistical analyses of panels **(C, D)** were performed by Two-way ANOVA with Tukey’s test for multiple comparisons. *p< 0.05. Statistical analysis of panel **(E)** was done by Log-Rank test. *p< 0.05; **p< 0.001.

Finally, we characterized the pathogenic abilities of the *maiA-1* strain with a set of *in vitro* and *in vivo* assays. First, we performed an *in vitro* characterization challenging mouse bone marrow-derived macrophages (BMMs) with our isogenic set of strains. No differences in phagocytosis rates were observed over 8 hours of co-culture between the BMMs and any of the three fungal strains (**Figure 9A**). These results could be related with the H_2_O_2_ susceptibility assay in which similar sized zones of clearance were found in all strains (**Figure 9B**). It is important to highlight that this assay was developed in GMM, condition in which pyomelanin production ability of the fungal strains (Af293, *maiA-1* and *maiA-1^comp.^*) is the same (**Figure 5B**). To test if pyomelanin production could have a protective effect against oxidative stress, the same assay was developed using GMM supplemented with 10 mM Phe and Tyr. As shown **Figure S7**, although a modest pyomelanin production can be observed (back of the plates) (**Figure S7B**), no statistical differences regarding the size of inhibition could be detected (**Figure S7A**).

In a detailed study of the BMM cells-pathogen interaction, significantly less TNF release was observed in the macrophages co-cultured with the *maiA-1* mutant strain compared to those co-cultured with either the wild-type or *maiA-1^comp.^* strain (**Figure 9C**). A very interesting finding was discovered when the β-glucan content of each fungal strain was studied. Specifically, the total glucan content was found to be significantly lower in the *maiA-1* mutant strain compared to the wild-type strain (**Figure 9D**). This result might indicate that reduced stimulation of BMMs induced by the *maiA-1* mutant strain could be attributable to its lower β-glucan content.

Moreover, the impact of the absence of the *maiA* gene on virulence was studied using a neutropenic mouse model of invasive aspergillosis (IA) (**Figure 9E**). The results revealed that 85% and 90% of the mice infected with the Af293 strain and the *maiA-1^comp.^*, respectively, had died after 15 days post-infection. In contrast, the group of mice infected with the *maiA-1* strain showed 40% survival at the end of the assay. The overall statistical analysis of the assay showed a significantly lower lethality of the *maiA-1* mutant strain.

**Figure 10:**
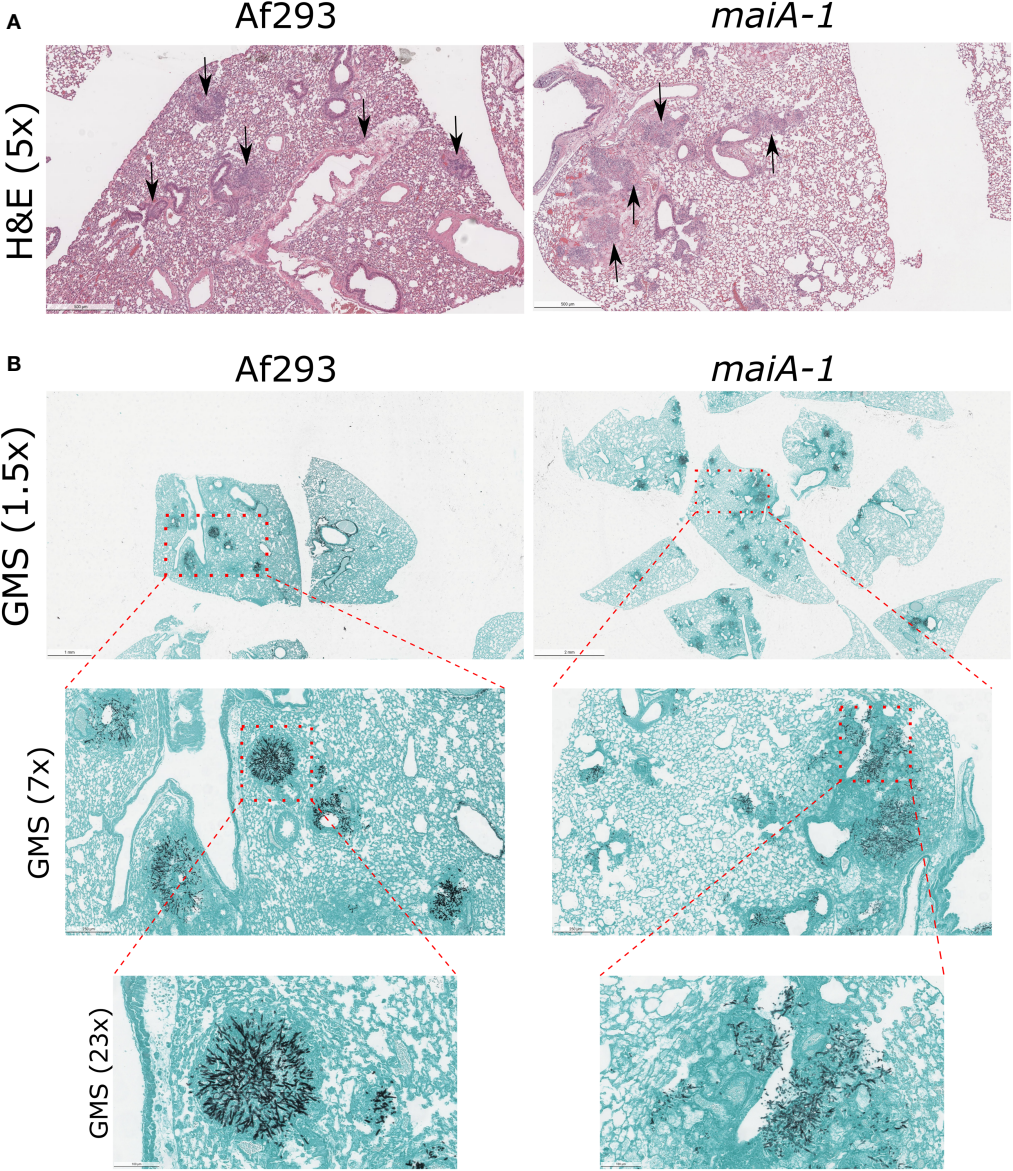
Histological study of mice infected with Af293 and *maiA-1* strain after 3 days post-infection. **(A)** Lung sections stained with Hematoxylin – Eosin (H&E). Pictures were taken with 5x magnification after 3 days post-infection. Black arrows indicate inflammation foci through the lung tissue. **(B)** Lung sections stained with Grocott’s Methenamine Silver (GMS). In the figure is possible to see consecutive magnifications from 1.5x to 23x of the same section of the lungs. Red squares highlight the area amplify in the next picture. The most representative pictures were shown in the figure.

This lower lethality showed by the *maiA-1* strain is not explained by the ability of this mutant to establish infection in lungs. As seen in **Figure 10**, no overt differences in fungal lesion presentation were found between those mice infected with the Af293 and the *maiA-1* strain at 3 days post-infection. Regarding the inflammation produced by the strains (**Figure 10A**), the lungs from mice infected with the Af293 strain presented a slightly higher inflammation homogeneously distributed through the organ than those lungs infected with the *maiA-1* strain. In relation with the GMS images (**Figure 10B**), is easy to see multiple *A. fumigatus* foci of both strains growing in the lungs after 3 days post-infection (1.5 magnification), but as more magnifications the pictures were taken, is easier to find more Af293 foci infecting the lung parenchyma than the *maiA-1* fungal strains that is more located around the airways and less in the parenchyma. Either way, these slight differences found between both fungal strains during the early stages of infection establishment likely do not explain the mortality differences.

## Discussion

In this work, RAW 264.7 macrophages and A549 human lung epithelial cells were used as *in vitro* infection models in co-incubation with *A. fumigatus* to perform two independent transcriptomic studies. After characterization of both models during co-incubation, when *A. fumigatus* reached 30% germination, the total mRNA was isolated and hybridize with the AWAFUGE 1.0 microarray. The transcriptomic analysis of both *in vitro* experimental assays showed that 140 A*. fumigatus* were up-regulated (FC > 1.5) in common in the two models used. To further prioritize the number of genes of interest, they were then compared with those detected as up-regulated during a previously published murine intranasal infection (Guruceaga et al., 2018). Using this approach, 13 genes were found to be up-regulated in all three abovementioned infection procedures. Some of these genes have been previously studied by other authors and have been related with the infection process or detected as expressed at the onset of the contact between cells or sera. Among them, it is important to highlight genes such as *aspf1*, that codifies an *A. fumigatus* allergen commonly found in different proteomic/transcriptomic studies (Guruceaga et al., 2018; Ramirez-Garcia et al., 2018) or the fumarate reductase Osm1, whose expression has been previously observed during hypoxia and during the first contact between the dormant conidia and the host (Teutschbein et al., 2010; Vödisch et al., 2011). Two up-regulated genes codify TCA cycle enzymes: the succinate dehydrogenase Sdh1 and the citrate synthase Cit1. The former protein has been previously described as expressed after human neutrophil exposure (Sugui et al., 2008), during the exit of the dormancy (Lamarre et al., 2008) as well as under hypoxia (Vödisch et al., 2011). Regarding the Cit1, some researchers have detected its expression during the contact with sera (Asif et al., 2010) and with airway epithelial cells (Oosthuizen et al., 2011); in addition, the cit1 gene was also described as essential for IA manifestation (Ibrahim-Granet et al., 2007).

The *A. fumigatus* catabolic Phe degradation pathway is encoded by a cluster of 6 genes (*hppD*, *hmgX*, *hmgA*, *fahA*, *maiA*, and *hmgR*) in this microorganism (Schmaler-Ripcke et al., 2009; Perez-Cuesta et al., 2020). Except for two genes (*fahA* and *maiA*), the cluster of genes involved in this pathway has been previously studied through the development of loss-of-function mutant strains. The Δ*hppD* null mutant displayed a lack of pyomelanin synthesis because HGA formation was blocked. Furthermore, the hyphae of this mutant showed increased susceptibility to hydrogen peroxide and other oxidizing agents when compared with Af293 (Schmaler-Ripcke et al., 2009). Moreover, this gene has been detected as overexpressed at 96 hours post-intranasal infection in comparison with the first 24 hours in immunosuppressed mice (Guruceaga et al., 2018). Other authors also detected the overexpression of *hpdA*, the *hppD* homolog in *Penicillium marneffei*, after exposure to macrophages (Boyce et al., 2015). In addition, the Δ*hmgX* mutant strain has been shown to have similarities with a Δ*hppD* strain (Keller et al., 2011). This Δ*hmgX* mutant was unable to produce HGA and, therefore, HmgX could be a cofactor or mediator of HppD enzyme function (Keller et al., 2011). On the other hand, the Δ*hmgA* mutant strain showed an increase in pyomelanin owing to the accumulation of HGA and displays reduced susceptibility to oxidizing agents compared to Δ*hppD* (Schmaler-Ripcke et al., 2009). Finally, the last gene of the Phe degradation pathway studied was the transcription factor *hmgR*. The Δ*hmgR* mutant is incapable of using Tyr as the sole carbon source or nitrogen source and, therefore, only a residual accumulation of pyomelanin was observed in cultures (Keller et al., 2011).

The only published study concerning *maiA* in *A. fumigatus* showed that this gene was overexpressed when the medium was supplemented with Tyr (Keller et al., 2011). Orthologs of this gene has been silenced in both *Aspergillus nidulans* and *P. marneffei* (Fernández-Cañón and Peñalva, 1995; Fernández-Cañón and Peñalva, 1998; Boyce et al., 2015). The catabolic Phe degradation pathway, in which *maiA* is putatively involved, is responsible for the production of one type of soluble melanin, known as pyomelanin, when an accumulation of intermediate metabolites, such as HGA or 4-MA, occurs. The mutation of the *maiA* gene is predicted to result in truncation of the final part of the pathway inhibiting the degradation of Phe or Tyr to fumarate and acetoacetate, which are destined to the Krebs cycle or the biosynthesis of other amino acids. Consequently, there would be an accumulation of 4-MA and HGA and the subsequent production of pyomelanin.

In accordance with previous publications (Schmaler-Ripcke et al., 2009; Keller et al., 2011; Perez-Cuesta et al., 2020), in response to Phe and Tyr, the *maiA-1* mutant strain would block the Phe degradation pathway causing a probable accumulation of 4-MA and, likely, of the previous metabolites of the pathway that can polymerizate to pyomelanin. Specifically, the mutant strain *maiA-1* started to produce this pigment when Phe and, especially, Tyr were added to the medium as the only carbon source. These results support our hypothesis that *A. fumigatus* can uses in a more effective way Phe than Tyr as an alternative carbo source. However, results obtained by other investigators using the Δ*hmgA* mutant showed that this strain produced more pyomelanin by the addition of Phe than Tyr (Schmaler-Ripcke et al., 2009).

Regarding basal growth analysis, our results suggest that the absence of the *maiA* gene did not impact on germination and minimally impacted on basic radial growth of the colonies. In contrast, the cell wall stress assays carried out in this study pointed out that the *maiA-1* strain suffers an alteration of the cell wall stability, which makes it unable to grow in presence of CR or CW, perhaps due to the lower content of glucans detected in its wall. Although, CR and CW are compounds with a chemical structure that can interact with β-linked glucans and, specifically, it is thought that both compounds interfere with cell wall assembly by binding to chitin, no differences in chitin content were detected between the fungal strains. Conceivably, CW and CR act by binding to nascent chitin chains, thereby inhibiting the assembly enzymes that connect chitin to β-1,3-glucan and β-1,6-glucan (Herth, 1980; Wood, 1980; Pringle, 1991). The ability to grow in the presence of both compounds was recovered by osmotic stabilization using sorbitol.

Other studies performed with mutant strains related with high-osmolarity glycerol response pathway (*shoA*, *msbA*, *opyA*, *sakA* and *mpkC*) which reported similar cell wall phenotypes (i.e., more sensitive to cell wall stress) are also more sensitive to osmotic stresses (Mattos et al., 2020; Silva et al., 2020) and to caspofungin. In contrast, the *maiA-1* strain displayed the same growth ability as the Af293 strain in the presence of osmotic stresses such as KCl or NaCl. In addition, the *maiA-1* strain showed normal susceptibility to the antifungal drugs tested (voriconazole, fluconazole, posaconazole, caspofungin and micafungin) following the EUCAST standardized method (data not shown). It is true that, while the *maiA* gene is involved in Phe degradation pathway, the other aforementioned genes are involved in the high-osmolarity glycerol response pathway and, therefore, the studies are not totally comparable. Another possible explanation is that, although the *maiA-1* mutant strain displayed a cell wall defect, this is not caused through an issue in the cell wall integrity pathway because this mutant strain was not affected by the presence of caffeine in the media as other investigators have previously described (Valiante et al., 2008; Valiante et al., 2009). This issue could be generated by a blockage of the Phe degradation pathway since the *maiA-1* strain is unable to complete the catabolic process of Phe and Tyr by which the fungus could synthesize new amino acids for structural purposes.

The SEM analysis showed a general unstructured appearance of the *maiA-1* mutant strain confirming a cell wall issue as we hypothesized from the CR and CFW stress assays, that is not related with the amount or distribution of chitin, exposed chitin or mannans in the cell wall. The Af293 hyphae looked similar to other SEM images presented by other investigators previously (Wuren et al., 2014; González-Ramírez et al., 2016; Joubert et al., 2017). However, the *maiA-1* hyphae showed differences in the external structure of the cell wall and a lack of a putative matrix-like substance that surrounded the apex of Af293 hyphae, although no differences in transcriptional levels of *uge3* were detected and in consequence a lack of galactosaminogalactan (Gravelat et al., 2013).

The last step of the research was to study the role of the *maiA* gene in fungal virulence. The cell wall is the main fungal target recognized by the immune cells and, for that, an *in vitro* model of infection using primary BMMs was performed. The *maiA-1* and Af293 strains showed the same H_2_O_2_ resistance ability, similar to the results previously described using Δ*hppD* and Δ*hmgA* mutants (Schmaler-Ripcke et al., 2009). Besides, Boyce and coworkers performed the same experiment with *P. marneffei* also found that Δ*hppA* and Δ*hmgA* showed a very mild sensitivity to H_2_O_2_ (Boyce et al., 2015). No other studies have previously described a similar phenotype with alterations in cell wall, and β-glucans composition, upon silencing genes involved in the Phe degradation pathway in *A. fumigatus*, *A. nidulans* or *P. marneffei*. However, this result could explain, not only why the *maiA-1* strain produced significantly less TNF release by BMMs after 16 hours, but also the phenotype showed in response to CR and CFW and the SEM images.

The lower mortality observed in neutropenic mice infected with the mutant strain is novel and interesting because other mutant strain of a gene involve in the Phe degradation pathway (Δ*hppD*) did not show any difference in the mortality rate observed when was compared with its complemented strain (*hppDc*) (Keller et al., 2011). In addition, it is important to note that these other studies used only transient immune suppression with corticosteroids instead of our model (cyclophosphamide and triamcinolone acetonide) that employs persistently immunosuppressed mice. What we can conclude about the lower mortality of the *maiA-1* strain is that is not caused by an issue in the establishment of the infection; more studies focused on how loss of *maiA* can modulate or impacts host-pathogen response are needed to understand how this gene interferes with the mortality ability.

In this study, an involvement of *maiA* with fungal cell wall, which is seemingly independent of classical MAPK regulation, has been established. Furthermore, a new Phe/Tyr fitness theory by which the use of Phe as sole carbon source is energetically more beneficial to *A. fumigatus* is proposed. Finally, the role of the *maiA* gene in fungal virulence has been revealed using a chemotherapeutically immune suppressed murine infection model. In fact, this is the first study in which a mutation of one of the genes involved in the Phe degradation pathway is directly related to *A. fumigatus* virulence. The process by which the *maiA-1* strain suffers cell wall defects should be studied in future investigations, but the impossibility of this mutant strain to utilize these amino acids in a more efficient way could be involved in the phenotype observed both related with cell wall defects as well as the decreased virulence.

## Data availability statement

The datasets presented in this study can be found in online repositories. The names of the repository/repositories and accession number(s) can be found below: https://www.ebi.ac.uk/arrayexpress/, E-MTAB-5314, E-MTAB-13476, A-MEXP-2352.

## Ethics statement

The animal study was approved by LACU committee of the University of Tennessee Health Science Center (Protocol number 22-0373). The study was conducted in accordance with the local legislation and institutional requirements.

## Author contributions

XG: Conceptualization, Investigation, Methodology, Writing – original draft, Writing – review & editing. UP: Investigation, Methodology, Writing – review & editing. AM: Investigation, Methodology, Writing – review & editing. EP: Investigation, Methodology, Writing – review & editing. HT: Investigation, Methodology, Writing – review & editing. SC: Investigation, Methodology, Writing – review & editing. JX: Investigation, Methodology, Writing – review & editing. AN: Investigation, Methodology, Writing – review & editing. AR: Conceptualization, Supervision, Writing – review & editing. JF: Funding acquisition, Supervision, Writing – review & editing. AR: Writing – review & editing, Conceptualization, Funding acquisition, Project administration, Supervision.

## Supplementary material

Supplementary Figure 1 Phenotypic spotting assay characterization of Af293, *maiA-1*, Δ*maiA*, and *maiA-1^comp^* using GMM agar plates supplemented with the indicated agents after 72 hours of incubation.

Supplementary Figure 2 Phenotypic characterization of Δ*maiA* in response to Phe and Tyr to demonstrate that the phenotype of whole deletion of *maiA* gene is the same as the disruption strain *maiA-1*. Pyomelanin secretion ability of the Af293 and Δ*maiA-1* growing in A) GMM broth, B) GMM broth supplemented with 10 mM Phe and C) GMM broth supplemented with 10 mM Tyr. D) Spotting assay after 72 hours of incubation of the Af293 and Δ*maiA* strains on salt agar plates in which the only carbon source were Phe (50 mM), Tyr (50 mM) or Phe and Tyr (50 mM each). GMM or SMM 6-well plates supplemented with different concentrations (0, 20, 40, 60, 80, 120, 160 µg/ml) of congo red (CR) (E) or calcofluor white (CFW) (F). Statistical analyses were performed by t-test. *p < 0.05.

Supplementary Figure 3 Schematic representation of the Phe degradation pathway as well as the metabolic pathways in which Phe or Tyr take part. Red boxes indicate metabolic pathways still un-described in *A. fumigatus*. The pink box indicates that the pathway is described in *A. fumigatus* but do not generate energy as consequence. Green boxes represent metabolic pathways by which *A. fumigatus* could obtain energy. Orange boxes represent the two mechanisms of *A. fumigatus* to obtain Phe. Grey boxes represent steps described in other species but not in *A. fumigatus*. Grey boxes and dashed grey lines are related to Tyr while continuous grey lines are related to Phe. This figure was built in based on KEGG pathway database (www.genome.jp/kegg/pathway.html).

Supplementary Figure 4 Loss of *maiA* function does not cause an evident loss of chitin synthase activity or abundance of chitin. A) GMM 6 well plates supplemented with different concentrations (0, 0.5, 1, 2, 4, 8 µg/ml) of Nikkomycin Z were inoculated using 1 x 10^3^ fresh conidia of each strain (Af293, *maiA-1*, and *maiA-1^comp.^*). The plates were incubated at 37°C for 48 h, time in which pictures were taken. B) Chitin detection during growth on solid media. 1 x 10^3^ fresh conidia of each strain were grown on solid media for 16-24 hours at 37°C. Every 2 hours a 150 µl of CFW stock solution (10 µg/ml in PBS) was used to quantify chitin by fluorescence detection. The data shown are the slope after plotting CFW fluorescence against biomass measured as absorbance at 600 nm. The results are the average ± SEM (n=16). C) Fluorescent micrographs taken after 16 hours culture at 37°C and stained with a CFW stock solution (1 µg/ml in PBS) for 10 minutes in dark. Samples were taken using a *Nikon Eclipse Ti2* microscope. To make easy the comparison of fluorescence between samples all the pictures were taken the same day and using 2 milli seconds of exposition time. In panels A and C, representative images of 3 replicates per condition are shown.

Supplementary Figure 5 Loss of *maiA* function does not impact the amount and distribution of exposed chitin or mannans in the cell wall. Fluorescent micrographs taken after 16 hours culture at 37°C and stained with a mix of CFW and WGA-Alexa Fluor 633 conjugate (A), or ConA-Alexa Fluor 647 conjugate (B). In panel A, we first show chitin staining with CFW to watch the whole hyphae, then exposed chitin stained with WGA and finally the co-localization of both after merging both images. To make easy the comparison of fluorescence between samples panel A pictures were taken the same day and using 2 milli seconds of exposition time for the CFW and 20 milli seconds for WGA, and panel B pictures were taken using 9 milli seconds of exposition time. Representative images of 3 replicates per condition are shown.

Supplementary Figure 6 Expression levels of *uge3* and *pksP* genes in the Af293, *maiA-1*, and *maiA-1^comp.^* fungal strains.

Supplementary Figure 7 Response of the fungal strains to H_2_O_2_ oxidative stress in absence or presence of Phe and Tyr. A) Inhibition halus diameter of a culture of the three fungal strains on GMM agar plates or GMM^+^ (GMM supplemented with 10 mM Phe & Tyr) after 48 hours of incubation in presence of 200 mM H_2_O_2_ in a central well of the plate. To do that, 1 x 10^7^ fresh conidia of each strain were seeded and spread over a GMM or GMM^+^ agar plates. After that, 50 µL of a 200 mM solution of H_2_O_2_ was added into a central well previously generated. The plates were incubated at 37°C for 48 h. Two-way ANOVA with Tukey’s test for multiple comparisons was applied to analyze the data but no statistical differences were found. **B)** Most representative pictures of the conditions explained in panel (**A**).

## References

[B1] Al Abdallah Q. Ge W. Fortwendel J. R. (2017). A simple and universal system for gene manipulation in *Aspergillus fumigatus: in vitro*-assembled Cas9-Guide RNA ribonucleoproteins coupled with microhomology repair templates. mSphere 2, 1–14. doi: 10.1128/msphere.00446-17 PMC5700375 29202040

[B2] Asif A. R. Oellerich M. Amstrong V. W. Gross U. Reichard U. (2010). Analysis of the cellular *Aspergillus fumigatus* proteome that reacts with sera from rabbits developing an acquired immunity after experimental aspergillosis. Electrophoresis 31, 1947–1958. doi: 10.1002/elps.201000015 20564691

[B3] Bayry J. Beaussart A. Dufrêne Y. F. Sharma M. Bansal K. Kniemeyer O.. (2014). Surface structure characterization of *Aspergillus fumigatus* conidia mutated in the melanin synthesis pathway and their human cellular immune response. Infection Immun. 82, 3141–3153. doi: 10.1128/IAI.01726-14 PMC4136205 24818666

[B4] Beauvais A. Latgé J. (2015). *Aspergillus* biofilm in *vitro* and in *vivo* . Microbiol Spectrum 3 (4), MB-0017-2015. doi: 10.1128/microbiolspec.MB-0017-2015 26350307

[B5] Boyce K. J. McLauchlan A. Schreider L. Andrianopoulos A. (2015). Intracellular growth is dependent on tyrosine catabolism in the dimorphic fungal pathogen *Penicillium marneffei* . PloS Pathog. 11, 1–30. doi: 10.1371/journal.ppat.1004790 PMC4374905 25812137

[B6] Braga G. U. L. Rangel D. E. N. Fernandes É.K.K. Flint S. D. Roberts D. W. (2015). Molecular and physiological effects of environmental UV radiation on fungal conidia. Curr. Genet. 61, 405–425. doi: 10.1007/s00294-015-0483-0 25824285

[B7] Cánovas D. Studt L. Marcos A. T. Strauss J. (2017). High-throughput format for the phenotyping of fungi on solid substrates. Sci. Rep. 7, 4289. doi: 10.1038/s41598-017-03598-9 28655890 PMC5487330

[B8] Chai L. Y. A. Netea M. G. Sugui J. Vonk A. G. Van De Sande W. W. J. Warris A.. (2010). *Aspergillus fumigatus* conidial melanin modulates host cytokine response. Immunobiology 215, 915–920. doi: 10.1016/j.imbio.2009.10.002 19939494 PMC2891869

[B9] Dichtl K. Helmschrott C. Dirr F. Wagener J. (2012). Deciphering cell wall integrity signalling in *Aspergillus fumigatus*: identification and functional characterization of cell wall stress sensors and relevant Rho GTPases: Cell wall integrity sensors and relevant Rho GTPases of *Aspergillus fumigatus* . Mol. Microbiol. 83, 506–519. doi: 10.1111/j.1365-2958.2011.07946.x 22220813

[B10] Eisenman H. C. Casadevall A. (2012). Synthesis and assembly of fungal melanin. Appl. Microbiol. Biotechnol. 93, 931–940. doi: 10.1007/s00253-011-3777-2 22173481 PMC4318813

[B11] Fernández-Cañón J. M. Peñalva M. A. (1995). Fungal metabolic model for human type I hereditary tyrosinaemia. Proc. Natl. Acad. Sci. United States America 92, 9132–9136. doi: 10.1073/pnas.92.20.9132 PMC40938 7568087

[B12] Fernández-Cañón J. M. Peñalva M. A. (1998). Characterization of a fungal maleylacetoacetate isomerase gene and identification of its human homologue. J. Biol. Chem. 273, 329–337. doi: 10.1074/jbc.273.1.329 9417084

[B13] Fortwendel J. R. Juvvadi P. R. Pinchai N. Perfect B. Z. Alspaugh J. A. Perfect J. R.. (2009). Differential effects of inhibiting chitin and 1,3-β-D-glucan synthesis in Ras and calcineurin mutants of *Aspergillus fumigatus* . Antimicrobial Agents Chemother. 53, 476–482. doi: 10.1128/AAC.01154-08 PMC2630655 19015336

[B14] Gastebois A. Clavaud C. Aimanianda V. Latgé J. P. (2009). *Aspergillus fumigatus*: cell wall polysaccharides, their byosinthesis and organization. Future Microbiol. 4, 583–595. doi: 10.2217/fmb.09.29 19492968

[B15] González-Ramírez A. I. Ramírez-Granillo A. Medina-Canales M. G. Rodríguez-Tovar A. V. Martínez-Rivera M. A. (2016). Analysis and description of the stages of *Aspergillus fumigatus* biofilm formation using scanning electron microscopy. BMC Microbiol. 16, 1–13. doi: 10.1186/s12866-016-0859-4 27756222 PMC5069814

[B16] Gow N. A. R. Latgé J.-P. Munro C. A. (2017). The fungal cell wall: structure, biosynthesis, and function. Microbiol. Spectr. 5, 1–25. doi: 10.1128/9781555819583.ch12 PMC11687499 28513415

[B17] Gravelat F. N. Beauvais A. Liu H. Lee M. J. Snarr B. D. Chen D.. (2013). *Aspergillus* galactosaminogalactan mediates adherence to host constituents and conceals hyphal β-glucan from the immune system. PloS Pathog. 9, e1003575. doi: 10.1371/journal.ppat.1003575 23990787 PMC3749958

[B18] Grice C. M. Bertuzzi M. Bignell E. M. (2013). Receptor-mediated signaling in *Aspergillus fumigatus* . Front. Microbio. 4. doi: 10.3389/fmicb.2013.00026 PMC3576715 23430083

[B19] Guruceaga X. Ezpeleta G. Mayayo E. Sueiro-Olivares M. Abad-Diaz-De-Cerio A. Urízar J. M. A.. (2018). A possible role for fumagillin in cellular damage during host infection by *Aspergillus fumigatus* . Virulence 9, 1548–1561. doi: 10.1080/21505594.2018.1526528 30251593 PMC6177242

[B20] Guruceaga X. Perez-Cuesta U. Pellon A. Cendon-Sanchez S. Pelegri-Martinez E. Gonzalez O.. (2021). *Aspergillus fumigatus* fumagillin contributes to host cell damage. JoF 7, 936. doi: 10.3390/jof7110936 34829223 PMC8619997

[B21] Heinekamp T. Thywißen A. Macheleidt J. Keller S. Valiante V. Brakhage A. A. (2013). *Aspergillus fumigatus* melanins: interference with the host endocytosis pathway and impact on virulence. Front. Microbiol. 3. doi: 10.3389/fmicb.2012.00440 PMC3548413 23346079

[B22] Herth W. (1980). Calcofluor white and congo red inhibit chitin microfibril assembly of poterioochromonas: Evidence for a gap between polymerization and microfibril formation. J. Cell Biol. 87, 442–450. doi: 10.1083/jcb.87.2.442 7430250 PMC2110758

[B23] Hillmann F. Novohradská S. Mattern D. J. Forberger T. Heinekamp T. Westermann M.. (2015). Virulence determinants of the human pathogenic fungus *Aspergillus fumigatus* protect against soil amoeba predation. Environ. Microbiol. 17, 2858–2869. doi: 10.1111/1462-2920.12808 25684622

[B24] Ibe C. Munro C. A. (2021). Fungal cell wall Proteins and signaling pathways form a cytoprotective network to combat stresses. JoF 7, 739. doi: 10.3390/jof7090739 34575777 PMC8466366

[B25] Ibrahim-Granet O. Dubourdeau M. Latgé J.-P. Ave P. Huerre M. Brakhage A. A.. (2007). Methylcitrate synthase from *Aspergillus fumigatus* is essential for manifestation of invasive aspergillosis. Cell Microbiol 10, 134–148. doi: 10.1111/j.1462-5822.2007.01025.x 17973657

[B26] Jahn B. Koch A. Schmidt A. Wanner G. Gehringer H. Bhakdi S.. (1997). Isolation and characterization of a pigmentless-conidium mutant of *Aspergillus fumigatus* with altered conidial surface and reduced virulence. Infect. Immun. 65, 5110–5117. doi: 10.1128/iai.65.12.5110-5117.1997 9393803 PMC175736

[B27] Jahn B. Langfelder K. Schneider U. Schindel C. Brakhage A. A. (2002). PKSP-dependent reduction of phagolysosome fusion and intracellular kill of *Aspergillus fumigatus* conidia by human monocyte-derived macrophages. Cell. Microbiol. 4, 793–803. doi: 10.1046/j.1462-5822.2002.00228.x 12464010

[B28] Joubert L. M. Ferreira J. A. Stevens D. A. Nazik H. Cegelski L. (2017). Visualization of *Aspergillus fumigatus* biofilms with scanning electron microscopy and variable pressure-scanning electron microscopy: a comparison of processing techniques. J. Microbiol. Methods 132, 46–55. doi: 10.1016/j.mimet.2016.11.002 27836634

[B29] Keller S. Macheleidt J. Scherlach K. Schmaler-Ripcke J. Jacobsen I. D. Heinekamp T.. (2011). Pyomelanin formation in *Aspergillus fumigatus* requires HmgX and the transcriptional activator HmgR but is dispensable for virulence. PloS One 6, e26604. doi: 10.1371/journal.pone.0026604 22046314 PMC3203155

[B30] Koenig S. Rühmann B. Sieber V. Schmid J. (2017). Quantitative assay of β-(1,3)–β-(1,6)–glucans from fermentation broth using aniline blue. Carbohydr. Polymers 174, 57–64. doi: 10.1016/j.carbpol.2017.06.047 28821106

[B31] Lamarre C. Sokol S. Debeaupuis J.-P. Henry C. Lacroix C. Glaser P.. (2008). Transcriptomic analysis of the exit from dormancy of *Aspergillus fumigatus* conidia. BMC Genomics 9, 417. doi: 10.1186/1471-2164-9-417 18796135 PMC2556354

[B32] Langfelder K. Jahn B. Gehringer H. Schmidt A. Wanner G. Brakhage A. A. (1998). Identification of a polyketide synthase gene (pksP) of *Aspergillus fumigatus* involved in conidial pigment biosynthesis and virulence. Med. Microbiol. Immunol. 187, 79–89. doi: 10.1007/s004300050077 9832321

[B33] Latgé J. Chamilos G. (2019). *Aspergillus fumigatus* and aspergillosis in 2019. Clin. Microbiol. Rev. 33, e00140–e00118. doi: 10.1128/CMR.00140-18 31722890 PMC6860006

[B34] López-Romero P. (2011). Pre-processing and differential expression analysis of Agilent microRNA arrays using the AgiMicroRna Bioconductor library. BMC Genomics 12, 64. doi: 10.1186/1471-2164-12-64 21269452 PMC3037903

[B35] Luther K. Torosantucci A. Brakhage A. A. Heesemann J. Ebel F. (2007). Phagocytosis of *Aspergillus fumigatus* conidia by murine macrophages involves recognition by the dectin-1 beta-glucan receptor and Toll-like receptor 2. Cell Microbiol. 9, 368–381. doi: 10.1111/j.1462-5822.2006.00796.x 16953804

[B36] Martin-Vicente A. Souza A. C. O. Al Abdallah Q. Ge W. Fortwendel J. R. (2018). SH3-class Ras guanine nucleotide exchange factors are essential for *Aspergillus fumigatus* invasive growth. Cell. Microbiol. 21, e13013. doi: 10.1111/cmi.13013 PMC6522298 30698898

[B37] Mattos E. C. Silva P. Valero C. Castro A. D. Taft C. A. Al-furaiji N.. (2020). The *Aspergillus fumigatus* phophoproteome reveals roles of high-osmolarity glycerol mitogen-activated protein kinases in promoting cell wall damage and caspofungin tolerance. mBio 11, e02962–e02919. doi: 10.1128/mBio.02962-19 PMC7002344 32019798

[B38] Oosthuizen J. L. Gomez P. Ruan J. Hackett T. L. Moore M. M. Knight D. A.. (2011). Dual organism transcriptomics of airway epithelial cells interacting with conidia of *Aspergillus fumigatus* . PloS One 6, e20527. doi: 10.1371/journal.pone.0020527 21655222 PMC3105077

[B39] Pal A. K. Gajjar D. U. Vasavada A. R. (2013). DOPA and DHN pathway orchestrate melanin synthesis in *Aspergillus* species. Med. Mycol. 52, 1–9. doi: 10.3109/13693786.2013.826879 23998343

[B40] Pellon A. Ramirez-Garcia A. Guruceaga X. Zabala A. Buldain I. Antoran A.. (2018). Microglial immune response is impaired against the neurotropic fungus *Lomentospora prolificans* . Cell. Microbiol. 20, e12847. doi: 10.1111/cmi.12847 29582549

[B41] Perez-Cuesta U. Aparicio-Fernandez L. Guruceaga X. Martin-Souto L. Abad-Diaz-de-Cerio A. Antoran A.. (2020). Melanin and pyomelanin in *Aspergillus fumigatus*: from its genetics to host interaction. Int. Microbiol. 23, 55–63. doi: 10.1007/s10123-019-00078-0 31020477

[B42] Priebe S. Kreisel C. Horn F. Guthke R. Linde J. (2015). FungiFun2: a comprehensive online resource for systematic analysis of gene lists from fungal species. Bioinformatics 31, 445–446. doi: 10.1093/bioinformatics/btu627 25294921 PMC4308660

[B43] Pringle J. R. (1991). Staining of bud scars and other cell wall chitin with calcofluor. Methods Enzymol. 194, 732–735. doi: 10.1016/0076-6879(91)94055-H 2005820

[B44] Ramirez-Garcia A. Pellon A. Buldain I. Antoran A. Arbizu-Delgado A. Guruceaga X.. (2018). Proteomics as a tool to identify new targets against *Aspergillus* and *Scedosporium* in the context of cystic fibrosis. Mycopathologia 183, 273–289. doi: 10.1007/s11046-017-0139-3 28484941

[B45] Rocha M. C. Fabri J. H. T. M. Simões I. T. Silva-Rocha R. Hagiwara D. Da Cunha A. F.. (2020). The cell wall integrity pathway contributes to the early stages of *Aspergillus fumigatus* asexual development. Appl. Environ. Microbiol. 86, e02347–e02319. doi: 10.1128/AEM.02347-19 32005734 PMC7082570

[B46] Samantaray S. Neubauer M. Helmschrott C. Wagener J. (2013). Role of the guanine nucleotide exchange factor Rom2 in cell wall integrity maintenance of *Aspergillus fumigatus* . Eukaryot. Cell 12, 288–298. doi: 10.1128/EC.00246-12 23264643 PMC3571289

[B47] Schmaler-Ripcke J. Sugareva V. Gebhardt P. Winkler R. Kniemeyer O. Heinekamp T.. (2009). Production of pyomelanin, a second type of melanin, via the tyrosine degradation pathway in *Aspergillus fumigatus* . Appl. Environ. Microbiol. 75, 493–503. doi: 10.1128/AEM.02077-08 19028908 PMC2620705

[B48] Silva L. P. Frawley D. de Assis L. J. Tierney C. Fleming A. B. Bayram O.. (2020). Putative membrane receptors contribute to activation and efficient signaling of mitogen-activated protein kinase cascades during adaptation of *Aspergillus fumigatus* to different stressors and carbon sources. mSphere 5, e00818–e00820. doi: 10.1128/msphere.00818-20 PMC7494837 32938702

[B49] Smyth G. K. (2004). Linear models and empirical bayes methods for assessing differential expression in microarray experiments. Stat. Appl. Genet. Mol. Biol. 3, 1–25. doi: 10.2202/1544-6115.1027 16646809

[B50] Souza A. C. O. Al Abdallah Q. DeJarnette K. Martin-Vicente A. Nywening A. V. DeJarnette C.. (2019). Differential requirements of protein geranylgeranylation for the virulence of human pathogenic fungi. Virulence 10, 511–526. doi: 10.1080/21505594.2019.1620063 31131706 PMC6550545

[B51] Strycker B. D. Han Z. Bahari A. Pham T. Lin X. Shaw B. D.. (2021). Raman characterization of fungal DHN and DOPA melanin biosynthesis pathways. JoF 7, 841. doi: 10.3390/jof7100841 34682262 PMC8540899

[B52] Sugui J. A. Kim H. S. Zarember K. A. Chang Y. C. Gallin J. I. Nierman W. C.. (2008). Genes differentially expressed in conidia and hyphae of *Aspergillus fumigatus* upon exposure to human neutrophils. PloS One 3, e2655. doi: 10.1371/journal.pone.0002655 18648542 PMC2481287

[B53] Teutschbein J. Albrecht D. Pötsch M. Guthke R. Aimanianda V. Clavaud C.. (2010). Proteome profiling and functional classification of intracellular proteins from conidia of the human-pathogenic mold *Aspergillus fumigatus* . J. Proteome Res. 9, 3427–3442. doi: 10.1021/pr9010684 20507060

[B54] Tsai H. F. Chang Y. C. Washburn R. G. Wheeler M. H. Kwon-Chung K. J. (1998). The developmentally regulated *alb1* gene of *Aspergillus fumigatus*: its role in modulation of conidial morphology and virulence. J. Bacteriol. 180, 3031–3038. doi: 10.1128/jb.180.12.3031-3038.1998 9620950 PMC107801

[B55] Valiante V. Heinekamp T. Jain R. Härtl A. Brakhage A. A. (2008). The mitogen-activated protein kinase MpkA of *Aspergillus fumigatus* regulates cell wall signaling and oxidative stress response. Fungal Genet. Biol. 45, 618–627. doi: 10.1016/j.fgb.2007.09.006 17981060

[B56] Valiante V. Jain R. Heinekamp T. Brakhage A. A. (2009). The MpkA MAP kinase module regulates cell wall integrity signaling and pyomelanin formation in *Aspergillus fumigatus* . Fungal Genet. Biol. 46, 909–918. doi: 10.1016/j.fgb.2009.08.005 19715768

[B57] Valiante V. Macheleidt J. Föge M. Brakhage A. A. (2015). The *Aspergillus fumigatus* cell wall integrity signaling pathway: drug target, compensatory pathways, and virulence. Front. Microbiol. 06. doi: 10.3389/fmicb.2015.00325 PMC4399325 25932027

[B58] Vödisch M. Scherlach K. Winkler R. Hertweck C. Braun H.-P. Roth M.. (2011). Analysis of the *Aspergillus fumigatus* proteome reveals metabolic changes and the activation of the Pseurotin A biosynthesis gene cluster in response to hypoxia. J. Proteome Res. 10, 2508–2524. doi: 10.1021/pr1012812 21388144 PMC3091480

[B59] Wood P. J. (1980). Specificity in the interaction of direct dyes with polysaccharides. Carbohydr. Res. 85, 271–287. doi: 10.1016/S0008-6215(00)84676-5

[B60] Wuren T. Toyotome T. Yamaguchi M. Takahashi-Nakaguchi A. Muraosa Y. Yahiro M.. (2014). Effect of serum components on biofilm formation by *Aspergillus fumigatus* and other *Aspergillus* species. Japanese J. Infect. Dis. 67, 172–179. doi: 10.7883/yoken.67.172 24858605

[B61] Yelton M. M. Hamer J. E. Timberlake W. E. (1984). Transformation of *Aspergillus nidulans* by using a *trpC* plasmid. Proc. Natl. Acad. Sci. United States America 81, 1470–1474. doi: 10.1073/pnas.81.5.1470 PMC344858 6324193

