# Supplementary figures and images for: The Aspergillus fumigatus maiA gene contributes to cell wall homeostasis and fungal virulence
Source: Front Cell Infect Microbiol. 2024 Jan 26;14:1327299. doi: 10.3389/fcimb.2024.1327299 (PMC10853476; doi:10.3389/fcimb.2024.1327299)

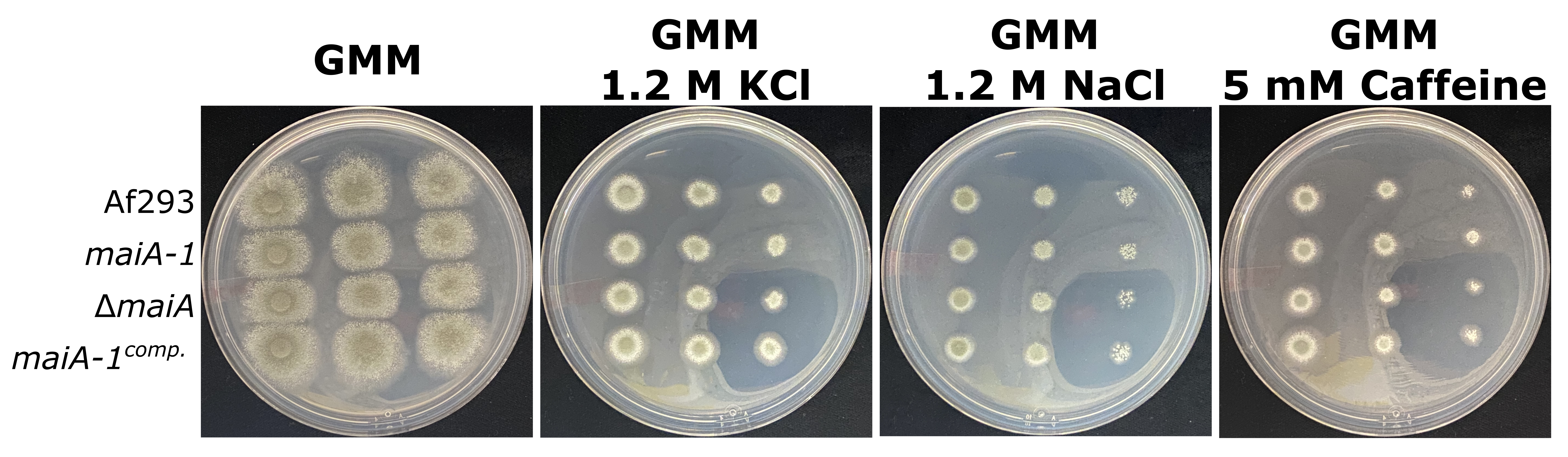

Supplement: Supplementary Figure 1 — Phenotypic spotting assay characterization of Af293, maiA-1, ΔmaiA, and maiA-1comp using GMM agar plates supplemented with the indicated agents after 72 hours of incubation. [file Image_1.tiff]

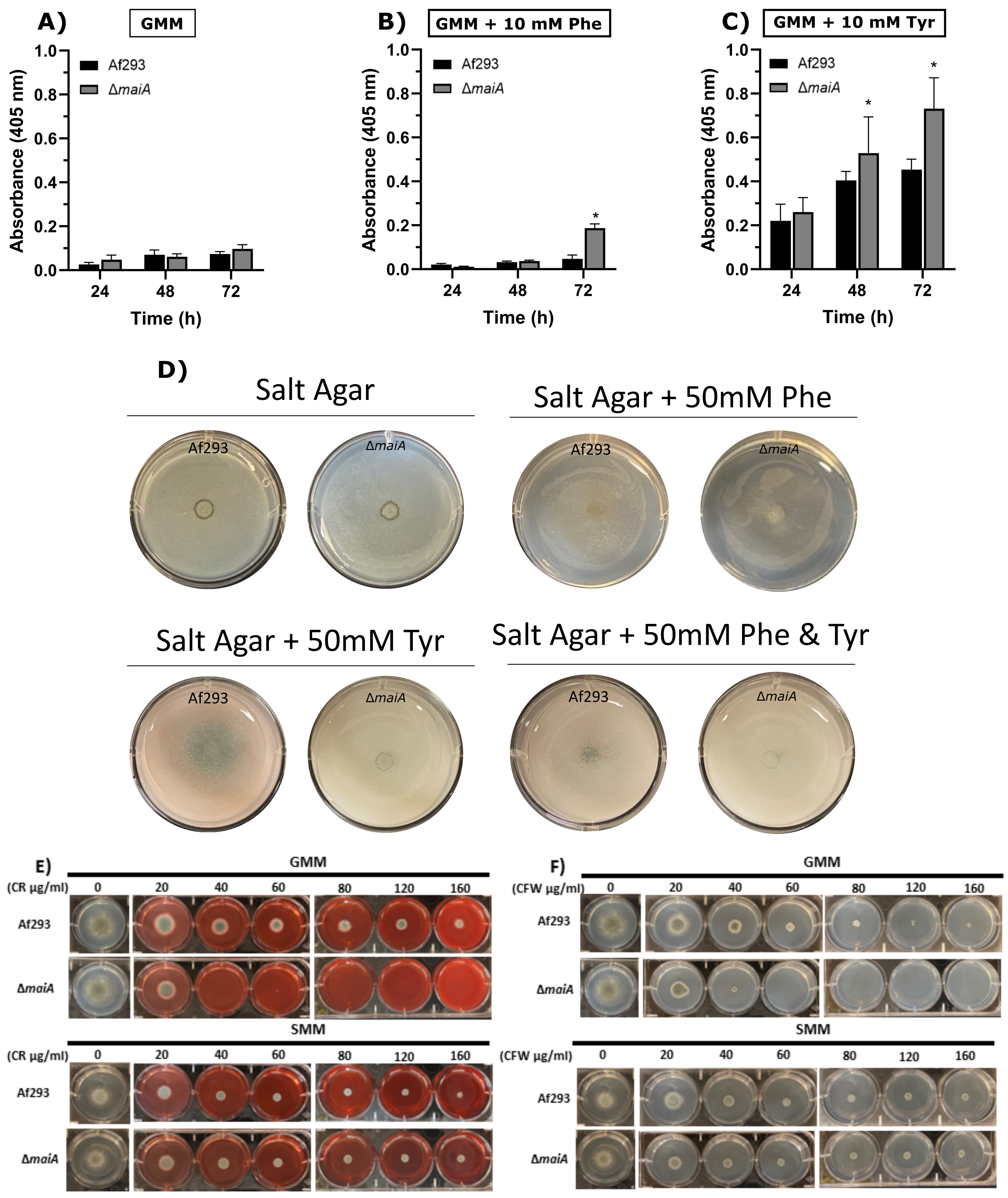

Supplement: Supplementary Figure 2 — Phenotypic characterization of ΔmaiA in response to Phe and Tyr to demonstrate that the phenotype of whole deletion of maiA gene is the same as the disruption strain maiA-1. Pyomelanin secretion ability of the Af293 and ΔmaiA-1 growing in A) GMM broth, B) GMM broth supplemented with 10 mM Phe and C) GMM broth supplemented with 10 mM Tyr. D) Spotting assay after 72 hours of incubation of the Af293 and ΔmaiA strains on salt agar plates in which the only carbon source were Phe (50 mM), Tyr (50 mM) or Phe and Tyr (50 mM each). GMM or SMM 6-well plates supplemented with different concentrations (0, 20, 40, 60, 80, 120, 160 µg/ml) of congo red (CR) (E) or calcofluor white (CFW) (F). Statistical analyses were performed by t-test. *p < 0.05. [file Image_2.tiff]

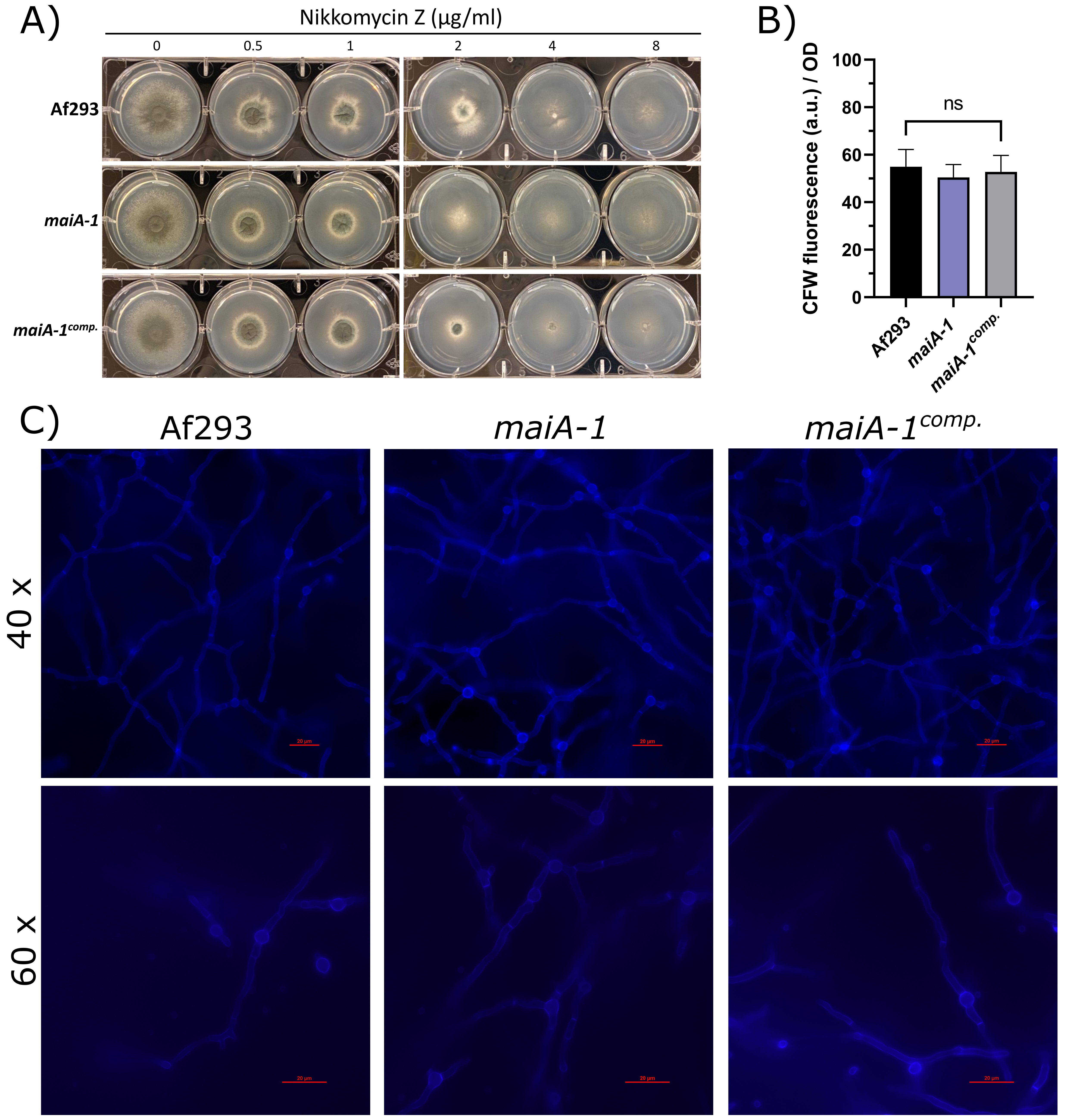

Supplement: Supplementary Figure 4 — Loss of maiA function does not cause an evident loss of chitin synthase activity or abundance of chitin. A) GMM 6 well plates supplemented with different concentrations (0, 0.5, 1, 2, 4, 8 µg/ml) of Nikkomycin Z were inoculated using 1 x 10^3^ fresh conidia of each strain (Af293, maiA-1, and maiA-1comp. ). The plates were incubated at 37°C for 48 h, time in which pictures were taken. B) Chitin detection during growth on solid media. 1 x 10^3^ fresh conidia of each strain were grown on solid media for 16-24 hours at 37°C. Every 2 hours a 150 µl of CFW stock solution (10 µg/ml in PBS) was used to quantify chitin by fluorescence detection. The data shown are the slope after plotting CFW fluorescence against biomass measured as absorbance at 600 nm. The results are the average ± SEM (n=16). C) Fluorescent micrographs taken after 16 hours culture at 37°C and stained with a CFW stock solution (1 µg/ml in PBS) for 10 minutes in dark. Samples were taken using a Nikon Eclipse Ti2 microscope. To make easy the comparison of fluorescence between samples all the pictures were taken the same day and using 2 milli seconds of exposition time. In panels A and C, representative images of 3 replicates per condition are shown. [file Image_4.tiff]

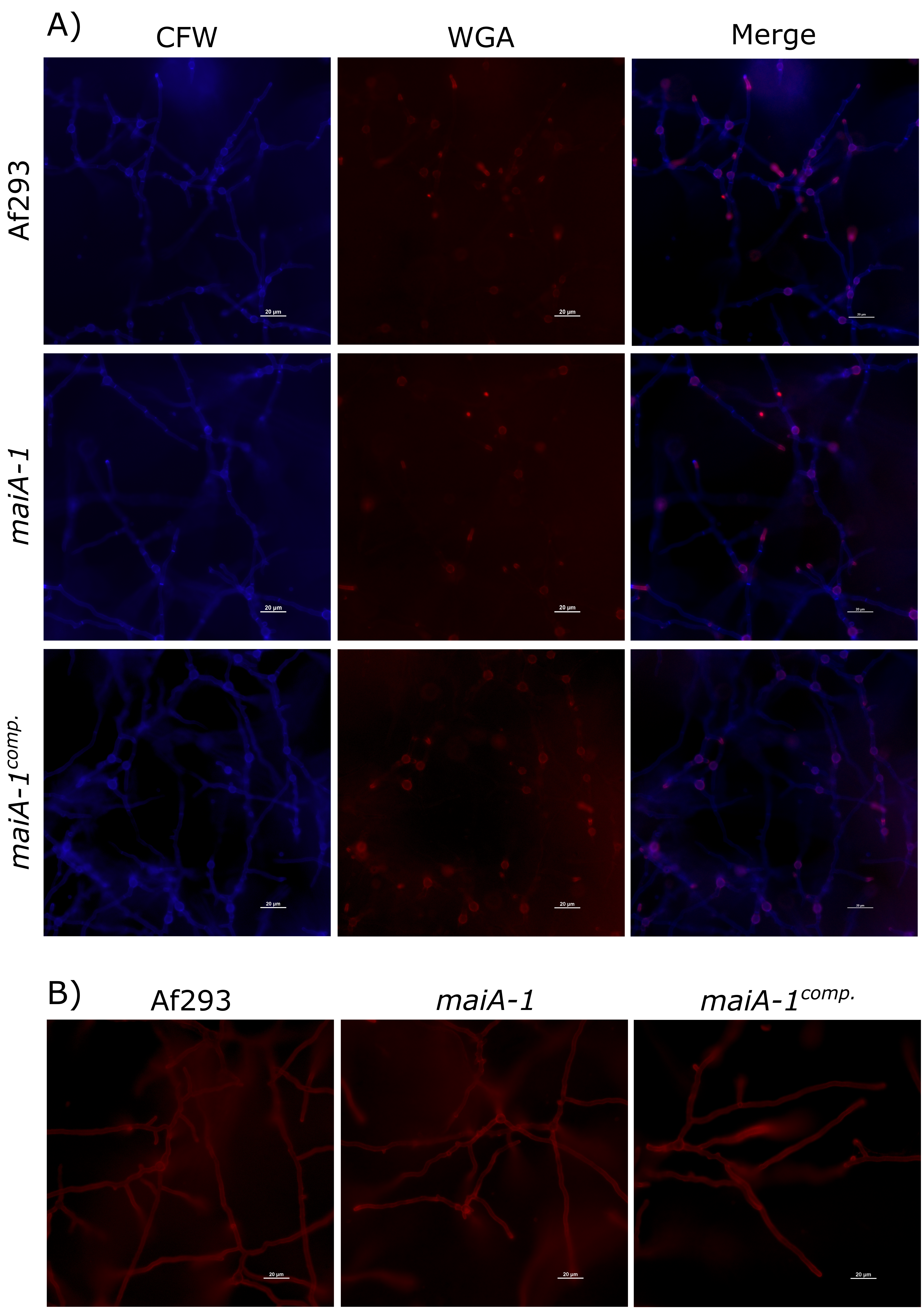

Supplement: Supplementary Figure 5 — Loss of maiA function does not impact the amount and distribution of exposed chitin or mannans in the cell wall. Fluorescent micrographs taken after 16 hours culture at 37°C and stained with a mix of CFW and WGA-Alexa Fluor 633 conjugate (A), or ConA-Alexa Fluor 647 conjugate (B). In panel A, we first show chitin staining with CFW to watch the whole hyphae, then exposed chitin stained with WGA and finally the co-localization of both after merging both images. To make easy the comparison of fluorescence between samples panel A pictures were taken the same day and using 2 milli seconds of exposition time for the CFW and 20 milli seconds for WGA, and panel B pictures were taken using 9 milli seconds of exposition time. Representative images of 3 replicates per condition are shown. [file Image_5.tiff]
