# Supplementary material for: The Aspergillus fumigatus maiA gene contributes to cell wall homeostasis and fungal virulence
Source: Front Cell Infect Microbiol. 2024 Jan 26;14:1327299. doi: 10.3389/fcimb.2024.1327299 (PMC10853476; doi:10.3389/fcimb.2024.1327299)
Supplement: Supplementary file 8 [file Table_1.docx]

**Table S1.** List of primers used for the microarray verification process.

| **Target^a^** | **Symbol^b^** | **Primer name^c^** | **Sequence (5´->3´) ^d^** | **Tm^e^** |
| --- | --- | --- | --- | --- |
| Aspartate aminotransferase | Afu1g04160 | 1g04160F | AGAGTCCCCAAGTAACGAGC | 59,10 |
|  |  | 1g04160R | CGTTGTGCGCTCATAATCGA | 59,08 |
| Hypothetical protein | Afu1g10450 | 1g10450F | GACCAGAGAAACCATCGCAC | 58,92 |
|  |  | 1g10450R | AGGCCTCCAAAGACTCTTCC | 59,01 |
| Maleylacetoacetate isomerase MaiA | Afu2g04240 | 2g04240F | AATCCTTCCGGTACAGTCCC | 58.80 |
|  |  | 2g04240R | GATGGTCACGGGTTCTTTGG | 58.83 |
| Rho GTPase ModA | Afu2g05740 | 2g05740F | CCGCTGGTCAGGAGGATTAT | 58,95 |
|  |  | 2g05740R | GAGTTTCTCACGAACGGCAG | 59,22 |
| Hypothetical protein | Afu2g08820 | 2g08820F | TCCGATCTCATGGTCATCCC | 58,66 |
|  |  | 2g08820R | CGGTTTGACAGGATGCAGAG | 58,92 |
| Rho GTPase Rac | Afu3g06300 | 3g06300F | TGTCCTACCCTCAAACCGAC | 59,03 |
|  |  | 3g06300R | GATACTTGTGCGCTCGGATC | 58,87 |
| WSC domain protein | Afu3g07050 | 3g07050F | TGAAATCCTGACAGCACCCT | 58,93 |
|  |  | 3g07050R | AATGGAATGAAAACCGCCCC | 59,10 |
| Succinate dehydrogenase subunit Sdh1 | Afu3g07810 | 3g07810F | CTTGAGGGACCAGAGACGTT | 59,03 |
|  |  | 3g07810R | CAGAGCAGCGTTGATACCAC | 59,00 |
| Hypothetical protein | Afu3g13080 | 3g13080F | CTCAACGAAGAACCACAGCC | 59,13 |
|  |  | 3g13080R | CAACCAGGTGAGAACGCAAA | 58,98 |
| MFS myo-inositol transporter | Afu4g01560 | 4g01560F | GATCAAACGTCTCGGACTGC | 59,01 |
|  |  | 4g01560R | AATGTAGAGGGGCTGGCAAT | 59,07 |
| Hypothetical protein | Afu5g02320 | 5g02320F | CTGCGAACCGTTCTGATGAG | 59,01 |
|  |  | 5g02320R | AGCAACGGTAAATCTCCCCA | 59,01 |
| Rheb small monomeric GTPase RhbA | Afu5g05480 | 5g05480F | TCGCCACAGAGATCGTTGAT | 59,18 |
|  |  | 5g05480R | CTCTTCGCCCAATTGCCTAC | 58,98 |
| Hypothetical protein | Afu5g08800 | 5g08800F | CTTCGGTATCACAATGGGCG | 59,06 |
|  |  | 5g08800R | CGTTGGTGTGGAGGTTGTTT | 58,90 |
| 3-methylcrotonyl-CoA carboxylase, beta subunit (MccB) | Afu5g08940 | 5g08940F | GGAGCGCACTTCATTGAACT | 58,84 |
|  |  | 5g08940R | TTCGGACCCCATAACACCAA | 58,93 |
| Pectin lyase | Afu5g10170 | 5g10170F | TGGACTCTACTCCTCCTCGG | 59,45 |
|  |  | 5g10170R | CAAAGTAGTTGCCCTCGAGC | 58,92 |
| Pectin lyase | Afu5g10380 | 5g10380F | GGAAATGCCTCCCCTGTCTA | 58,79 |
|  |  | 5g10380R | CACGAGTTCTGGTTGATGGC | 59,20 |
| Succinyl-CoA:3-ketoacid-coenzyme A transferase (ScoT) | Afu6g12250 | 6g12250F | AAGGCTTTGGAGGTGCAATG | 59,03 |
|  |  | 6g12250R | CATGGGCTTGACATCATCCG | 59,05 |
| Lipase | Afu7g04020 | 7g04020F | TCGGAGCATTGCCTCCTTAA | 59,09 |
|  |  | 7g04020R | TGGAGCTTTGACCGGGTAAA | 59,23 |
| Methionine aminopeptidase type II (metAP) | Afu8g00410 | 8g00410F | TTCCCGAACAACTCCTATCC | 58.98 |
|  |  | 8g00410R | TTGTCGAGGTGACGTTTCTC | 58.85 |
| Phytanoil-CoA dioxygenase | Afu8g00480 | 8g00480F | TCTGGTTCCAACGTGACTGA | 58.88 |
|  |  | 8g00480R | CATGTGAGGCGCCGAATAAT | 58.77 |
| Secreted antimicrobial peptide | Afu8g00710 | 8g00710F | TGCTACCATCGTCTATCCCTAC | 58,58 |
|  |  | 8g00710R | TCTTGTAGTTGCCGCTCATG | 58,27 |
| Elastinolytic metalloproteinase Mep | Afu8g07080 | 8g07080F | TCCATGCTGTACGAGGTGTT | 59,03 |
|  |  | 8g07080R | CCTGGACAAAGTTGGGGTTG | 58,96 |
| 4-Hydroxyphenylpyruvate dioxygenase | Afu2g04200 | 2g04200F | TATGCAGCGACGAACAATGG | 58.99 |
|  |  | 2g04200R | CTAAAGTGTGCGTGGTCTCG | 58.94 |
| Aromatic aminotransferase Aro8 | Afu2g13630 | 2g13630F | TCCAGGTCTCATCTCGTTGG | 58,81 |
|  |  | 2g13630R | TCGGTATGCTCAGTCACGAA | 58,83 |
| Conidial pigment biosynthesis oxidase Arb2 | Afu2g17530 | 2g17530F | GCTATCAGTCCTGCTTCCGA | 59,25 |
|  |  | 2g17530R | GAGCTTGCGGACTCAGAATG | 58,99 |
| Conidial pigment biosynthesis scytalone dehydratase Arp1 | Afu2g17580 | 2g17580F | TGCGAAAGTGGGATGACATG | 58,55 |
|  |  | 2g17580R | GCGGTAGTAGTGCTCATTGG | 58,43 |
| Conidial pigment polyketide synthase PksP/Alb1 | Afu2g17600 | 2g17600F | GGTCAATGGAGTGGCTCTTAC | 62,00 |
|  |  | 2g17600R | CCCTGGTACTCTGGTTTGTATTT | 62,00 |
| Catalase Cat | Afu2g18030 | 2g18030F | GTCGAACAACTGGCCTTCTC | 58,85 |
|  |  | 2g18030R | CGTCACGAAGAGTTGGGTTG | 59,14 |
| Aromatic-L-amino-acid decarboxylase | Afu3g02240 | 3g02240F | TTACATCACGCTTGGCATGG | 58,91 |
|  |  | 3g02240R | AAACTGTCAACACCTTCCGC | 58,98 |
| Phospholipase D1 (PLD1) | Afu3g05630 | 3g05630F | CGATCCAACTCCAGTGCTTG | 58,92 |
|  |  | 3g05630R | CTCGCTGTCAGGCATACAAC | 59,00 |
| Stress responsive A/B barrel domain protein | Afu4g10610 | 4g10610F | ACGCATCACTCTTTTCAACGT | 58,79 |
|  |  | 4g10610R | CGGCGCTGAGAATATACGGT | 60,04 |
| Allergen and cytotoxin AspF1 | Afu5g02330 | 5g02330F | CACGCCCATCAAATTCGGAA | 59.19 |
|  |  | 5g02330R | TCCTTGGGTTTCTTCGAGTCA | 58.96 |
| Rheb small monomeric GTPase RhbA | Afu5g05480 | 5g05480F | TCGCCACAGAGATCGTTGAT | 59,18 |
|  |  | 5g05480R | CTCTTCGCCCAATTGCCTAC | 58,98 |
| MAP kinase kinase kinase SteC | Afu5g06420 | 5g06420F | AGTGTATTGGTGGCCGAAGA | 59,02 |
|  |  | 5g06420R | TGAGCTCTCCAGTGATTGCA | 59,02 |
| GATA transcriptional activator AreA | Afu6g01970 | 6g01970F | ACCCAAGCGATTTCCCTACA | 59,01 |
|  |  | 6g01970R | GGCGTTGTCATCACCAAAGT | 59,05 |
| Cytochrome P450 monooxygenase | Afu6g02210 | 6g02210F | GGGTGTATCGGGAAGAGCAT | 59,24 |
|  |  | 6g02210R | TACTTGGCGCTCGTGAAATG | 58,92 |
| Citrate synthase Cit1 | Afu6g03590 | 6g03590F | TGTTTTGGCTGCTCTTGACG | 59,34 |
|  |  | 6g03590R | ACTTGATCAGGTCCAAAATATGC | 57,36 |
| Nonribosomal peptide synthase GliP | Afu6g09660 | 6g09660F | GTTGGACTGGGAATGCGTTT | 59,04 |
|  |  | 6g09660R | GCTTTCGTGAGTGACCGTAC | 58,94 |
| Indoleamine 2,3-dioxygenase family protein | Afu7g02010 | 7g02010F | CCTGAAGAGGTTCCCTCATATC | 61 |
|  |  | 7g02010R | GACGTGCAGTAGGGTAGAAAG | 62 |
| Defensin domain protein | Afu7g05180 | 7g05180F | ACAACTCCATCTCCTGCATGA | 59,09 |
|  |  | 7g05180R | CCACGGTATCCTCCTCGATG | 59,4 |
| Phospholipase PldA | Afu7g05580 | 7g05580F | GCGACAAACACGAGGTCAAT | 59,13 |
|  |  | 7g05580R | TGCCTTTTCACTCTCCGGAT | 59,02 |
| Hypothetical protein | Afu8g05100 | 8g05100F | GTAGCCAGCAAGTCACATCG | 59 |
|  |  | 8g05100R | CCGGAATACTAGAGGGCCTG | 59,03 |
| Fumarate reductase Osm1 | Afu8g05530 | 8g05530F | CCACAACATGCCCTTTGACA | 58,96 |
|  |  | 8g05530R | GCAAGTTCACCACAAGCGTA | 59,06 |
| Alpha/beta hydrolase | Afu2g02920 | H_AlphBe_F | ACTGGTCGTGACACTGTTGG | 59.62 |
|  |  | H_AlphBet_R | CGCATTGTGGATGCTAGACT | 58.90 |
| Mis12-Mtw1 family protein | Afu3g13950 | H_Mis12_F | GCTCTCGAATTGGTTTGGAC | 59.68 |
|  |  | H_Mis12_R | ATGTTTTTCGGGTTCGGTTT | 60.57 |
| Isochorismatase family protein | Afu3g14500 | H_Isochor_F | GAAGAACGACGGAGTGGTAAG | 62 |
|  |  | H_Isochor_R | CTCAGGTAGGTCCAAGTCATAAAT | 62 |
| Molybdopterin synthase small subunit CnxG | Afu7g01580 | H_Molybdop_F | ACCAGTCTTCCAAATCCACTAC | 62 |
|  |  | H_Molybdop_R | TAGGAAATCAAAGAGCCTGGAC | 62 |
| Glucose-6-phosphate 1-dehydrogenase 2 | NM_01468.2 | MmF | CCTTTGGTACTGAGGGTCGT | 59.03 |
|  |  | MmR | ATCCATTGGCAGCTTCTCCT | 59.08 |

^a^Product description of the genes chosen to verify the microarray data following RefSeq nomenclature.

^b^Systematic name of the gene following AspGD nomenclature.

^c^F: Forward; R: Reverse

^d^Sequence of each primer.

^e^Tm: Melting temperature of each primer.
