# Supplementary material for: The Aspergillus fumigatus maiA gene contributes to cell wall homeostasis and fungal virulence
Source: Front Cell Infect Microbiol. 2024 Jan 26;14:1327299. doi: 10.3389/fcimb.2024.1327299 (PMC10853476; doi:10.3389/fcimb.2024.1327299)
Supplement: Supplementary file 9 [file Table_2.docx]

**Table S2.** List of primers and gRNAs used for the genetic manipulations.

| **Primer name** | **Sequence (5´->3´)^a^** | **Tm^b^** |
| --- | --- | --- |
| maiA_Dis_Hyg_Fw | TCTTTCATAATCAAAGTTAACATCGTCTTTGCATCCAACGagcttgcatgcctgcaggtc | 72.5 |
| maiA_Dis_Hyg_Rv | CAAAGTCACCCTATACACCTATTTCCGCTCCTCCTGCTCAcatcgatgatatcagatctt | 72.5 |
| maiA_Del_Hyg_Fw | TACCCAAGTGGCACTCTTTCATAATCAAAGTTAACATCGTagcttgcatgcctgcaggtc | 73 |
| maiA_Del_Hyg_Rv | AATGTGCACAAAGGAAGCCTTGTCCCAACATCTTAAACCAcatcgatgatatcagatctt | 71.4 |
| maiA_Del_Scr_Fw | CTCCGCATGTAAACGATCAG | 61.6 |
| maiA_Del_Scr_Rv | TCCAGCCCTAAAGTTGGAGT | 58.7 |
| maiA_Comp_Fw | TCTTTCATAATCAAAGTTAACATCGTCTTTGCATCCAACGatcaccATGGAAGACTCAACAAGCCC | 72.4 |
| maiA_Comp_Rv | AAGAGCTGATATGCATTCCAAATGTGCACAAAGGAAGCCTAAGCTTcggagaatatggag | 72.2 |
| Fw_Clon_BamHI | tttggatccATGGAAGACTCAACAAGCCC | 67.1 |
| Rv_Clon_SbfI | tttcctgcaggTCAAGAAGAACTCACTCGAA | 66.9 |
| Fw_qPCR_pksP | AAGACGTCTCGACATCCTCC | 58.9 |
| Rv_qPCR_pksP | TCCGGGAACATGAAGAGCTT | 59.1 |
| Fw_qPCR_uge3 | CAAACTGGCCGAGAACTTCC | 58.4 |
| Rv_qPCR_uge3 | GGGATGTGCTTGGAGGAAAC | 58.6 |
| gRNA5’ | TTTGCATCCAACGATCACCA | N/A |
| gRNA3’ | TGAGTTCTTCTTGAGACAAT | N/A |
| gRNA5'Comp | CAACGagcttgcatgcctgc | N/A |
| gRNA3'Comp | GACGCAGCCTGATACGCCGG | N/A |

^a^Sequence of each primer.

^b^Tm: Melting temperature of each primer.
