# Supplementary material for: The Aspergillus fumigatus maiA gene contributes to cell wall homeostasis and fungal virulence
Source: Front Cell Infect Microbiol. 2024 Jan 26;14:1327299. doi: 10.3389/fcimb.2024.1327299 (PMC10853476; doi:10.3389/fcimb.2024.1327299)
Supplement: Supplementary file 10 [file Table_3.docx]

**Table S3.** Go enrichment analysis of the *A. fumigatus* up-regulated genes (FC > 1.5) and down-regulated genes (FC < -1.5) in both *in vitro* experimental models of infection.

|  | **Number of Genes** | | | |
| --- | --- | --- | --- | --- |
|  | **RAW 264.7 *vs* Control** | | **A549 *vs* Control** | |
| **GO Slim Term** | **Up-regulated** | **Down-regulated** | **Up-regulated** | **Down-regulated** |
| **Biological process** | **126** | **101** | **403** | **189** |
| Transport | 35 | 34 | 89 | 40 |
| Regulation of biological process | 22 | 8 | 56 | 22 |
| Response to stress | 17 | 15 | 36 | 8 |
| Secondary metabolic process | 17 | 23 | 30 | 6 |
| Response to chemical | 14 | 17 | 40 | 11 |
| Lipid metabolic process | 13 | 8 | 30 | 3 |
| RNA metabolic process | 13 | 7 | 18 | 10 |
| Developmental process | 13 | 1 | 20 | 5 |
| Cellular amino acid metabolic process | 12 | 7 | 19 | 8 |
| Transcription, DNA-templated | 11 | 3 | 15 | 4 |
| Cellular respiration | 11 | - | 6 | 1 |
| Carbohydrate metabolic process | 10 | 20 | 49 | 23 |
| Toxin metabolic process | 9 | 2 | 12 | - |
| Cellular homeostasis | 7 | 18 | 9 | 7 |
| Cell cycle | 6 | 2 | 12 | 6 |
| Filamentous growth | 6 | 1 | 18 | 7 |
| Sexual sporulation | 6 | 1 | 7 | 2 |
| Asexual sporulation | 4 | - | 5 | 3 |
| Organelle organization | 3 | 1 | 16 | 11 |
| Pathogenesis | 3 | 7 | 12 | 4 |
| Translation | 2 | - | 1 | 2 |
| Cellular protein modification process | 2 | - | 9 | 10 |
| Conjugation | 1 | - | 3 | - |
| DNA metabolic process | 1 | - | 2 | 2 |
| Vitamin metabolic process | 1 | 2 | 1 | 2 |
| Cell adhesion | 1 | - | 3 | - |
| Signal transduction | 1 | - | 6 | 5 |
| Transposition | 1 | - | - | - |
| Ribosome biogenesis | - | 2 | 1 | 2 |
| Cytokinesis | - | 1 | 1 | 3 |
| Protein catabolic process | - | 1 | 2 | 4 |
| Cytoskeleton organization | - | - | 5 | 2 |
| Vesicle-mediated transport | - | - | 4 | 3 |
| Protein folding | - | - | 2 | - |
| Other | 35 | 34 | 105 | 52 |

|  | **Number of Genes** | | | |
| --- | --- | --- | --- | --- |
|  | **RAW 264.7 *vs* Control** | | **A549 *vs* Control** | |
| **GO Slim Term** | **Up-regulated** | **Down-regulated** | **Up-regulated** | **Down-regulated** |
| **Cellular component** | **161** | **160** | **536** | **259** |
| Membrane | 55 | 33 | 112 | 49 |
| Mitochondrion | 32 | 4 | 28 | 5 |
| Nucleus | 27 | 8 | 37 | 18 |
| Cytosol | 20 | 3 | 10 | 5 |
| Plasma membrane | 17 | 8 | 32 | 7 |
| Extracellular region | 13 | 12 | 38 | 16 |
| Vacuole | 8 | 3 | 8 | 3 |
| Endomembrane system | 7 | 3 | 10 | 12 |
| Cell wall | 6 | 1 | 11 | 1 |
| Peroxisome | 6 | 3 | 9 | - |
| Endoplasmic reticulum | 5 | - | 2 | 6 |
| Golgi apparatus | 3 | - | 3 | 2 |
| Site of polarized growth | 1 | - | 7 | 3 |
| Nucleolus | - | 1 | - | 2 |
| Cell cortex | - | - | 6 | 1 |
| Cytoskeleton | - | - | 4 | 3 |
| Chromosome | - | - | 2 | 5 |
| Actin cytoskeleton | - | - | 1 | 1 |
| Microtubule cytoskeleton | - | - | 1 | 2 |
| Ribosome | - | - | - | 2 |
| Other | 11 | 11 | 41 | 9 |

|  | **Number of Genes** | | | |
| --- | --- | --- | --- | --- |
|  | **RAW 264.7 *vs* Control** | | **A549 *vs* Control** | |
| **GO Slim Term** | **Up-regulated** | **Down-regulated** | **Up-regulated** | **Down-regulated** |
| **Molecular function** | **146** | **106** | **407** | **198** |
| Oxidoreductase activity | 62 | 37 | 117 | 54 |
| Hydrolase activity | 24 | 32 | 89 | 40 |
| Transporter activity | 21 | 18 | 48 | 13 |
| Transferase activity | 21 | 20 | 47 | 28 |
| DNA binding | 8 | 7 | 21 | 8 |
| Lyase activity | 8 | 2 | 14 | 5 |
| Peptidase activity | 7 | 1 | 10 | 5 |
| Protein binding | 3 | - | 8 | 2 |
| RNA binding | 2 | - | 5 | 4 |
| Isomerase activity | 2 | - | 5 | - |
| Protein kinase activity | 1 | - | 5 | - |
| Signal transducer activity | 1 | - | - | - |
| Structural molecule activity | 1 | - | - | - |
| Lipase activity | 1 | 1 | 4 | - |
| Phosphatase activity | 1 | - | 10 | 1 |
| Ligase activity | 1 | - | - | - |
| Enzyme regulator activity | 1 | 1 | - | - |
| Helicase activity | - | 1 | 1 | 2 |
| Ligase activity | - | - | 3 | 2 |
| Enzyme regulator activity | - | - | 3 | - |
| Nucleotidyltransferase activity | - | - | 1 | 1 |
| Structural molecule activity | - | - | 1 | 1 |
| Translation regulator activity | - | - | - | 1 |
| Other | 17 | 20 | 54 | 16 |
