# Supplementary material for: The Aspergillus fumigatus maiA gene contributes to cell wall homeostasis and fungal virulence
Source: Front Cell Infect Microbiol. 2024 Jan 26;14:1327299. doi: 10.3389/fcimb.2024.1327299 (PMC10853476; doi:10.3389/fcimb.2024.1327299)
Supplement: Supplementary file 11 [file Table_4.docx]

**Table S4.** List of common *A. fumigatus* up-regulated DEGs during the co-incubation of the fungus with the macrophages RAW 264.7 and the human lung epithelium cell line A549.

|  |  |  | **Fold Change (log_2_)** | |
| --- | --- | --- | --- | --- |
| **ID** | **Product** |  | **RAW 264.7 vs Control** | **A549 vs Control** |
| Afu1g01300 | GPI anchored protein |  | 4.663 | 2.609 |
| Afu1g03570 | Acid phosphatase PHOa | *phoA* | 2.228 | 7.182 |
| Afu1g04160 | Aspartate aminotransferase |  | 3.223 | 2.452 |
| Afu1g06610 | NADH-quinone oxidoreductase, 23 kDa subunit |  | 2.774 | 3.180 |
| Afu1g07380 | Glutamate synthase Glt1 |  | 2.110 | 1.544 |
| Afu1g13510 | C6 transcription factor FacB/Cat8 |  | 2.392 | 2.545 |
| Afu1g13750 | C_2_H_2_ transcription factor (Rpn4) |  | 1.663 | 2.095 |
| Afu1g14540 | Oxidoreductase, short-chain dehydrogenase/reductase family |  | 2.474 | 3.855 |
| Afu1g14550 | Mn superoxide dismutase MnSOD |  | 4.439 | 5.605 |
| Afu1g15590 | Succinate dehydrogenase subunit CybS |  | 2.927 | 1.708 |
| Afu1g17070 | FYVE domain protein |  | 2.640 | 3.023 |
| Afu1g17360 | bZIP transcription factor (BACH2) |  | 2.437 | 5.423 |
| Afu2g00630 | GDSL lipase/acylhydrolase family protein |  | 1.751 | 2.509 |
| Afu2g00890 | Conserved hypothetical protein |  | 1.553 | 2.057 |
| Afu2g02180 | Hypothetical protein |  | 1.565 | 1.343 |
| Afu2g02460 | Hypothetical protein |  | 2.462 | 4.392 |
| Afu2g03730 | Ctr copper transporter family protein |  | 2.334 | 3.116 |
| Afu2g04200 | 4-hydroxyphenylpyruvate dioxygenase |  | 6.384 | 6.783 |
| Afu2g04210 | Hypothetical protein |  | 3.843 | 4.234 |
| Afu2g04220 | Homogentisate 1,2-dioxygenase | *hmgA* | 3.782 | 3.726 |
| Afu2g04240 | Maleylacetoacetate isomerase | *maiA* | 2.416 | 2.219 |
| Afu2g04262 | C6 transcription factor |  | 2.331 | 1.539 |
| Afu2g05060 | Alternative oxidase AlxA |  | 4.657 | 5.252 |
| Afu2g07720 | Cytochrome b5 |  | 3.462 | 2.262 |
| Afu2g07910 | Myo-inositol transporter |  | 1.840 | 2.280 |
| Afu2g09400 | Cyclohexanone monooxygenase |  | 1.643 | 2.002 |
| Afu2g10540 | Hypothetical protein |  | 1.602 | 2.300 |
| Afu2g12630 | Allergenic cerato-platanin Asp f 13 | *aspf13* | 1.817 | 1.429 |
| Afu2g12710 | MFS monocarboxylate transporter. putative |  | 4.852 | 2.753 |
| Afu2g12780 | von Willebrand domain protein |  | 1.645 | 3.149 |
| Afu2g13175 | Hypothetical protein |  | 2.093 | 3.159 |
| Afu2g15860 | TAM domain methyltransferase |  | 3.356 | 1.866 |
| Afu2g16180 | Hypothetical protein |  | 1.616 | 1.682 |
| Afu2g16930 | Succinate:fumarate antiporter (Acr1) |  | 3.965 | 4.153 |
| Afu2g17790 | Amino acid transporter |  | 1.924 | 5.138 |
| Afu3g01150 | GPI anchored cell wall protein, putative |  | 3.154 | 2.034 |
| Afu3g03040 | Hypothetical protein |  | 2.144 | 3.023 |
| Afu3g03060 | Cell wall protein PhiA |  | 4.723 | 4.309 |
| Afu3g03230 | bZIP transcription factor |  | 1.760 | 1.932 |
| Afu3g03280 | FAD binding monooxygenase |  | 3.064 | 2.326 |
| Afu3g03290 | Hypothetical protein |  | 2.595 | 3.634 |
| Afu3g03310 | RTA1 domain protein |  | 3.383 | 4.797 |
| Afu3g06730 | MFS sugar transporter |  | 1.696 | 3.086 |
| Afu3g07810 | Succinate dehydrogenase subunit | *sdh1* | 2.721 | 1.519 |
| Afu3g07990 | GABA permease GabA |  | 1.622 | 4.559 |
| Afu3g08210 | Hypothetical protein |  | 1.729 | 2.506 |
| Afu3g09220 | P450 family fatty acid hydroxylase |  | 1.936 | 2.563 |
| Afu3g12920 | Non-ribosomal peptide synthase GliP-like |  | 2.194 | 2.061 |
| Afu3g13640 | Extracellular serine-rich protein |  | 1.779 | 2.691 |
| Afu3g13660 | Ctr copper transporter family protein |  | 5.135 | 4.005 |
| Afu3g13670 | Siderochrome-iron transporter |  | 4.181 | 2.705 |
| Afu3g13680 | Hypothetical protein |  | 4.238 | 3.022 |
| Afu3g13690 | Pyoverdine/dityrosine biosynthesis family protein, putative |  | 3.119 | 1.698 |
| Afu3g13700 | Transferase family protein |  | 6.523 | 5.245 |
| Afu3g14665 | Hypothetical protein |  | 4.943 | 4.108 |
| Afu3g14940 | Hypothetical protein |  | 2.490 | 2.713 |
| Afu4g01270 | Integral membrane protein |  | 3.144 | 1.868 |
| Afu4g01290 | Endo-chitosanase, pseudogene |  | 3.606 | 3.776 |
| Afu4g01470 | C6 finger domain protein |  | 2.026 | 2.629 |
| Afu4g03240 | Cell wall serine-threonine-rich galactomannoprotein Mp1 |  | 2.800 | 3.524 |
| Afu4g03270 | Epoxide hydrolase |  | 1.535 | 2.714 |
| Afu4g03410 | Flavohemoprotein |  | 4.829 | 3.099 |
| Afu4g03920 | MFS drug transporter |  | 3.569 | 5.353 |
| Afu4g03930 | Cysteine synthase B |  | 4.722 | 6.870 |
| Afu4g03940 | Ferric-chelate reductase |  | 3.165 | 3.276 |
| Afu4g04190 | Hypothetical protein |  | 3.355 | 4.413 |
| Afu4g04530 | Short chain dehydrogenase/reductase (Ayr1) | *ayr1* | 1.535 | 2.467 |
| Afu4g08490 | acyl-CoA dehydrogenase |  | 2.373 | 2.396 |
| Afu4g09110 | Cytochrome c peroxidase Ccp1 |  | 4.674 | 3.321 |
| Afu4g09140 | L-ornithine aminotransferase Car2 |  | 3.120 | 1.683 |
| Afu4g09470 | Cytochrome P450 monooxygenase |  | 2.532 | 3.903 |
| Afu4g09560 | ZIP Zinc transporter |  | 1.695 | 1.373 |
| Afu4g09580 | Major allergen Asp f 2 | *aspf2* | 2.676 | 2.452 |
| Afu4g10610 | Stress responsive A/B barrel domain protein |  | 1.591 | 1.942 |
| Afu4g10690 | Iron-sulfur cluster assembly accessory protein Isa1, putative |  | 3.122 | 2.066 |
| Afu4g13510 | Isocitrate lyase AcuD |  | 4.034 | 4.707 |
| Afu4g13540 | Potassium uptake transporter |  | 1.955 | 2.238 |
| Afu4g13780 | Polyphenol monooxygenase |  | 2.289 | 4.003 |
| Afu5g00300 | Zinc-binding oxidoreductase |  | 3.549 | 3.817 |
| Afu5g00740 | Hypothetical protein |  | 1.908 | 2.496 |
| Afu5g01030 | Glyceraldehyde 3-phosphate dehydrogenase |  | 1.927 | 3.091 |
| Afu5g01200 | Carboxypeptidase S1 |  | 2.858 | 2.204 |
| Afu5g02320 | Conserved hypothetical protein |  | 2.068 | 3.266 |
| Afu5g02330 | Major allergen and cytotoxin Asp f 1 (MitF) | *aspf1* | 3.001 | 3.102 |
| Afu5g07480 | Hypothetical protein |  | 1.705 | 1.695 |
| Afu5g07500 | β-lactamase family protein |  | 1.761 | 2.657 |
| Afu5g09330 | CipC-like antibiotic response protein |  | 2.636 | 4.687 |
| Afu5g10370 | Iron-sulfur protein subunit of succinate dehydrogenase | *sdh2* | 2.986 | 1.928 |
| Afu5g11260 | Siderophore transcription factor | *sreA* | 2.104 | 2.095 |
| Afu5g11290 | D-amino acid oxidase |  | 1.551 | 1.559 |
| Afu5g12840 | Hydroxyacylglutathione hydrolase |  | 2.209 | 2.194 |
| Afu5g13800 | Transcriptional regulator |  | 2.291 | 2.068 |
| Afu5g13810 | Transulfuration enzyme family protein |  | 2.494 | 3.435 |
| Afu5g14650 | RING finger protein |  | 1.662 | 3.628 |
| Afu6g00290 | Aminotransferase |  | 1.678 | 1.504 |
| Afu6g00430 | IgE-binding protein |  | 2.836 | 2.241 |
| Afu6g00680 | Conserved hypothetical protein |  | 2.285 | 2.038 |
| Afu6g00690 | Conserved hypothetical protein |  | 3.229 | 3.073 |
| Afu6g00720 | LysM domain protein |  | 1.989 | 2.779 |
| Afu6g00740 | Conserved hypothetical protein |  | 2.983 | 4.180 |
| Afu6g02210 | Cytochrome P450 monooxygenase |  | 2.459 | 3.678 |
| Afu6g02810 | Ctr copper transporter |  | 1.810 | 1.767 |
| Afu6g02820 | Metalloreductase |  | 1.760 | 1.903 |
| Afu6g03540 | Malate synthase | *acuE* | 2.848 | 3.706 |
| Afu6g03590 | Citrate synthase | *cit1* | 2.610 | 3.541 |
| Afu6g07710 | Mitocondrial dicarboxylate carrier protein |  | 1.858 | 3.376 |
| Afu6g07720 | Phosphoenolpyruvate carboxykinase | *acuF* | 3.707 | 3.663 |
| Afu6g07750 | MFS phospholipid transporter | *git1* | 2.105 | 2.506 |
| Afu6g09200 | Hypothetical protein |  | 2.230 | 1.594 |
| Afu6g10310 | Hypothetical protein |  | 2.313 | 3.249 |
| Afu6g12250 | Succinyl-CoA:3-ketoacid-coenzyme A transferase (ScoT), putative | *scoT* | 2.072 | 3.276 |
| Afu6g12930 | Mitochondrial aconitate hydratase |  | 2.077 | 2.090 |
| Afu6g13750 | Ferric-chelate reductase |  | 2.445 | 1.820 |
| Afu6g14010 | GPI anchored protein |  | 3.245 | 2.255 |
| Afu7g00990 | Transcriptional activator of ethanol catabolism AlcS |  | 4.067 | 4.992 |
| Afu7g01020 | Hypothetical protein |  | 2.092 | 1.221 |
| Afu7g01050 | Salicylate hydroxylase |  | 2.203 | 2.215 |
| Afu7g02010 | Indoleamine 2,3-dioxygenase family protein |  | 5.696 | 3.663 |
| Afu7g02070 | AIF-like mitochondrial oxidoreductase (Nfrl) |  | 2.957 | 3.208 |
| Afu7g05490 | Conserved hypothetical protein |  | 3.083 | 3.841 |
| Afu7g05500 | Glutathione S-transferase |  | 2.045 | 1.817 |
| Afu7g06180 | Hypothetical protein |  | 2.220 | 3.612 |
| Afu7g06380 | Maltase |  | 1.663 | 1.977 |
| Afu7g06680 | AAA family ATPase |  | 1.735 | 2.200 |
| Afu7g06820 | Galactose oxidase |  | 1.519 | 2.193 |
| Afu7g07020 | Hypothetical protein |  | 2.404 | 4.156 |
| Afu7g07060 | Hypothetical protein |  | 2.285 | 4.371 |
| Afu7g08310 | Hypothetical protein |  | 2.245 | 4.523 |
| Afu8g00790 | Hypothetical protein |  | 3.704 | 2.542 |
| Afu8g01310 | Ferric-chelate reductase | *fre2* | 6.239 | 6.378 |
| Afu8g01670 | Bifunctional catalase-peroxidase | *cat2* | 1.828 | 2.289 |
| Afu8g02050 | Conserved hypothetical protein |  | 2.061 | 3.535 |
| Afu8g02060 | Glycan biosynthesis protein (PigL), putative |  | 3.115 | 4.641 |
| Afu8g02070 | Glycosyl transferase |  | 3.893 | 5.660 |
| Afu8g02090 | Nucleotide-sugar transporter |  | 2.299 | 3.432 |
| Afu8g02620 | CobW domain protein |  | 2.871 | 6.542 |
| Afu8g05530 | Fumarate reductase | *osm1* | 2.522 | 2.555 |
| Afu8g05730 | β-glucosidase |  | 2.191 | 3.195 |
| Afu8g06080 | Flavohemoprotein |  | 4.032 | 3.217 |
